# Boosting Long-Term Stability of Pure Formamidinium Perovskite Solar Cells by Ambient Air Additive Assisted Fabrication

**DOI:** 10.1021/acsenergylett.1c01311

**Published:** 2021-09-13

**Authors:** K. M. Muhammed Salim, Sofia Masi, Andrés Fabián Gualdrón-Reyes, Rafael S. Sánchez, Eva M. Barea, Marie Kreĉmarová, Juan F. Sánchez-Royo, Iván Mora-Seró

**Affiliations:** † Institute of Advanced Materials (INAM), University Jaume I, Avenida de Vicent Sos Baynat, s/n, 12071 Castelló de la Plana, Castellón, Spain; ‡ Institute of Materials Science (ICMUV), University of Valencia, c/Catedrático José Beltrán, 2, 46980 Paterna, Valencia, Spain; § MATINÉE: CSIC Associated Unit (ICMM-ICMUV of the University of Valencia), Universidad de Valencia, Valencia, Spain

## Abstract

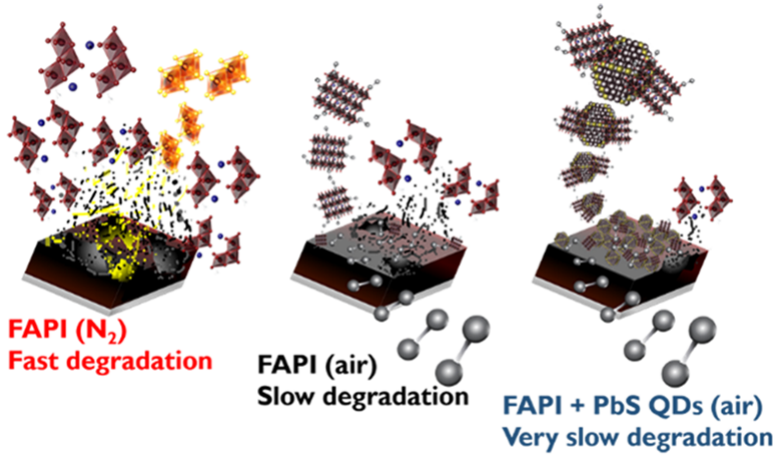

Due to the high industrial interest for perovskite-based photovoltaic devices, there is an urgent need to fabricate them under ambient atmosphere, not limited to low relative humidity (RH) conditions. The formamidinium lead iodide (FAPI) perovskite α-black phase is not stable at room temperature and is challenging to stabilize in an ambient environment. In this work, we show that pure FAPI perovskite solar cells (PSCs) have a dramatic increase of device long-term stability when prepared under ambient air compared to FAPI PSCs made under nitrogen, both fabricated with *N*-methylpyrrolidone (NMP). The *T*_80_ parameter, the time in which the efficiency drops to 80% of the initial value, increases from 21 (in N_2_) to 112 days (in ambient) to 145 days if PbS quantum dots (QDs) are introduced as additives in air-prepared FAPI PSCs. Furthermore, by adding methylammonium chloride (MACl) the power conversion efficiency (PCE) reaches 19.4% and devices maintain 100% of the original performance for at least 53 days. The presence of Pb–O bonds only in the FAPI films prepared in ambient conditions blocks the propagation of α- to δ-FAPI phase conversion. Thus, these results open the way to a new strategy for the stabilization in ambient air toward perovskite solar cells commercialization.

The recently emerged and astonishing halide perovskites (HPs) with an ABX_3_ composition [A = Cs^+^ (cesium), CH_3_NH_3_^+^ (methylammonium, MA), or NH_2_CH=NH_2_^+^ (formamidinium, FA); B = Pb^2+^ or Sn^2+^; X = Cl^–^, Br^–^, I^–^] have been recognized as one of the most promising next-generation photovoltaic materials.^1^ The prior works on single-junction halide perovskite solar cells (PSCs) have begun in 2009; then after successive progress has been made by allowing for a rapid improvement of the power conversion efficiencies (PCEs) from 3.8% to 25.5% over the past years.^2−4^ Apart from the solar cell applications, the HPs with a solid-state thin-film structure have been implemented in lasing,^5,6^ light-emitting diodes,^7−10^ photodetectors,^11−13^ X-ray detectors,^14^ etc. These take benefit for perovskite staggering properties like benign defect physics, low-cost solution processing, tunable bandgap, high absorption coefficient, low exciton binding energy, and good charge carrier mobility.^15^ Mainly, the initial research on HPs materials has been focused on methylammonium lead iodide (MAPI) due to its perovskite phase stability at room temperature,^16,17^ despite it presents a bandgap (1.55 eV) higher than the optimum for solar cell applications. Most of the works have reported on the preparation of this layer under an inert N_2_ atmosphere. However, the preparation of perovskite solar cells (PSCs) in ambient air conditions even at moderate/high relative humidity (RH) conditions will undoubtedly pave the way to its industrial development and commercialization. An improved photoluminescence (PL) of MAPI prepared under ambient conditions has been demonstrated,^18^ and air post-treatments^19,20^ have been exploited to improve the MAPI solar cells open-circuit voltage (*V*_oc_). The fortuitous or strategic exposure to the ambient atmosphere has led to a moisture-assisted intermediate, which has improved the performances of inherently stable perovskite.^21,22^ However, this kind of approach has been left out in the case of metastable perovskite, like the formamidinium perovskite,^23^ due to the fast phase transition from the black α-phase to the yellow δ-phase, boosted after exposure to oxygen and water. Nevertheless, the lower bandgap of 1.48 eV of formamidinium lead iodide (FAPI) than MAPI, closer to the ideal one determined by the Schockley-Queisser limit,^24^ has identified it as a superior candidate for single-junction solar cells.^25−27^ Important milestones have been achieved in terms of the stability of the formamidinium PSCs prepared under controlled conditions in the glovebox,^28−30^ but their fabrication in air has received less attention^31^ and mostly at low-moisture conditions not under real ambient conditions. Indeed, to switch on an ambient fabrication, a method strongly preferred for industrial production, the whole fabrication process and the growth dynamics of perovskite cubic phase must be well regulated allowing for better crystallinity and morphology. Yet it has been proved that the process under ambient atmosphere, even if it increases the grain size and the crystallinity of the perovskite, could reduce the film coverage.^32,33^

Interestingly, for different halide perovskites the demonstrated efficiency and stability were acceptable in comparison with devices prepared using similar procedures but under an inert atmosphere,^21,34,35^ boosting the interest to extend these studies to pure FAPI. For FAPI based PSCs, the well-known instability of the black phase triggered by moisture likely made that studies of fabrication in air atmosphere were made at low-RH (<20% or drybox) conditions. However, the most extended approach to enhance the stability of FAPI-based PSCs has been the introduction of additional cations. For instance, FA_0.85_Cs_0.15_PbI_3_ based solar cells fabricated at RH = 55% showed better thermal stability than the one fabricated under nitrogen;^36^ a slightly different composition of FA_0.90_Cs_0.10_PbI_3_ showed improvement in UV and moisture stability.^37^ The triple cation^38^ PSCs with a combination of Cs_0.05_(MA_0.17_FA_0.83_)_0.95_Pb(I_0.83_Br_0.17_)_3_ fabricated in the air with low-humidity conditions (RH< 25%) obtained a higher PCE of 20.8% than the same set of devices fabricated in a dry nitrogen atmosphere and preserved relatively high efficiency of 19.5% after 18 weeks under an RH of 20–35%.^38,39^ It is worth noting that in the case of mixed cation-halide perovskite exposed to light and humidity more pathways have been found leading to the ultimate degradation of the perovskite; one of the reasons is the phase separation issues distinguished in mixed cation-halide perovskite.^40^ In addition, the use in FAPI of alternative cations and anions to FA^+^ and I^–^, respectively, produces an increase of the bandgap with the subsequent decrease of the maximum theoretical efficiency. This vision highlights once again the interest in a pure FAPI long-term stabilization in ambient air. Consequently, in the past few years, the tendency in the record PSCs is to use compositions as close as possible to FAPI, also using air atmosphere with low/moderate RH. PSCs based on FA_0.92_MA_0.08_PbI_3_ with PEAI post-treatment and the fabrication in the air at 30–40% RH achieved a high PCE of 23.32%.^41^ Very recently, the anion engineering concept that uses the pseudohalide formate anion (HCOO^–^) to mitigate the anion-vacancy defects and the resulting PSCs with FA_0.95_MA_0.05_PbI_3_, where MA is introduced by the use of the extended additive methylammonium chloride (MACl), additive thus attained a certified PCE of 25.2%,^42^ fabricated in an air atmosphere at low RH of 20%. These works highlight the enormous interest in the pure FAPI phase stability of devices fabricated in the air atmosphere and more importantly the physical origin of this stability even at high RH for further optimization of PSCs.

Beyond the compositional engineering, the use of additives has been the second big approach for the fabrication of PSCs in air conditions. The additive strategies aid the formation of high-quality perovskite crystals and guarantee an ideal morphology, preventing or decelerating the moisture vulnerability.^43,44^ The most common Lewis acid–base approach in the case of FAPI, is based on the use of *N*-methyl pyrrolidone (NMP).^26^ The intermediate FAI·PbI_2_·NMP adduct^31,45^ assists the formation of smooth and pinhole-free perovskite thin films at ambient atmosphere, and the pure FAPI reaches a stabilized PCE of 16.69% stable for about 1 month at low RH < 20%. Another additive with several advantages is embedded PbS quantum dots (QDs) into the perovskite thin films.^46−49^ The chemi-structural match of PbS QDs^28^ or nanoplatelets (NPLs)^50^ with perovskite (MAPI or FAPI) crystal phase results in the generation of seed-like nucleation regions for the bulky-epitaxial growth of high-quality perovskite absorber with enhanced optoelectronic properties and improved long-term stability.^28,50−53^ Although the huge interest of FAPI PSCs advancement in the stabilization of the FAPI solar cells, and the pieces of evidence that NMP improves its stability, the foundation of the improvement of the formamidinium stabilization was not yet analyzed,^23,26^ especially beyond low RH in real ambient conditions including medium-high RH fabrication conditions and how these conditions could affect the final device stability. Second, the perovskite-based devices fabricated in air^21^ are not always compared with the one fabricated under nitrogen, and the stability tests were still carried out with storage under nitrogen or RH < 20%,^31,45^ so the investigation of the stability of devices fabricated in both conditions is highly demanding, to shine a light on the origin of the enhanced stabilization under ambient conditions of materials normally affected by humid conditions,^38,39^ in order to develop industrially friendly fabrication processes.

In this work, first we have analyzed PSCs stability in the most demanding conditions by using pure FAPI without including any additional cation or anion that blue shifts the FAPI bandgap. We stabilized the ambient air fabricated pure FAPI (black α-FAPI phase)^23^ perovskite without changing the spectral characteristics, by the benefits of synergistic interaction of halide perovskites with additives PbS QDs and of the NMP–perovskite adduct. We found that unencapsulated FAPI PSCs fabricated in the air (25 °C and RH 40–60%) using NMP are significantly more stable than the analogous cells fabricated under nitrogen presenting also higher PCE. The addition of PbS QDs increases further both PCS and long-term stability. The optimized unencapsulated devices employing FAPI-PbS QDs showcased improved stability, showing a *T*_80_ parameter, the time in which the PCE drops to the 80% of the initial value, of 145 days, in comparison with a *T*_80_ of 112 and 21 for devices fabricated without PbS QDs under ambient and N_2_ atmosphere, respectively. This significant long-term stability enhancement is correlated to the presence of Pb–O bonds in the α-FAPI perovskite film fabricated under air conditions in contrast with films fabricated under N_2_, corroborated by Raman spectroscopy and X-ray Photoelectron Spectroscopy (XPS) measurements. Pb–O bonds would block the propagation of phase transformation from α-FAPI into δ-FAPI phase. Finally, we observe the same trend but with enhanced PCE when MACl additive is used, observing an increase of performance for air fabricated samples especially when PbS QDs are used as additives in comparison with N_2_. An impressive champion performance of 19.4% has been obtained for devices fabricated in ambient conditions with PbS QDs and a high RH of 50–60%.

The solar cells with FAPI active layer have been fabricated as reference cells either under ambient conditions or under nitrogen atmosphere. The method adopted is based on the formation of the intermediate phase with the NMP;^31,45^ see the Experimental Section in the Supporting Information. In the present work, PSCs were fabricated under moderate/high RH conditions (25 °C, RH = 40–60%), higher than in the previous reports, using a flat solar cell configuration.^54^ The advantage of NMP is that it is effective in different humidity conditions and with different solvents and solvent ratios.^31,45^

Different optical properties have been observed for FAPI films prepared following the same procedure but in N_2_ or air atmosphere. The photoluminescence (PL) intensity is higher in the case of the perovskite prepared under air, pointing to lower nonradiative recombination,^18,34^Figure 1a. Lower PL from samples fabricated under N_2_ atmosphere in comparison with air fabricated is observed independently of the side of the perovskite film analyzed, Figure S1a. Note that both samples were measured at ambient condition and the observed quenching cannot be correlated with the quench produced for PL measured in N_2_ conditions,^55^Figure S1b, pointing to intrinsic film properties as the origin of the nonradiative recombination increase for N_2_ fabricated samples.

**Figure 1:**
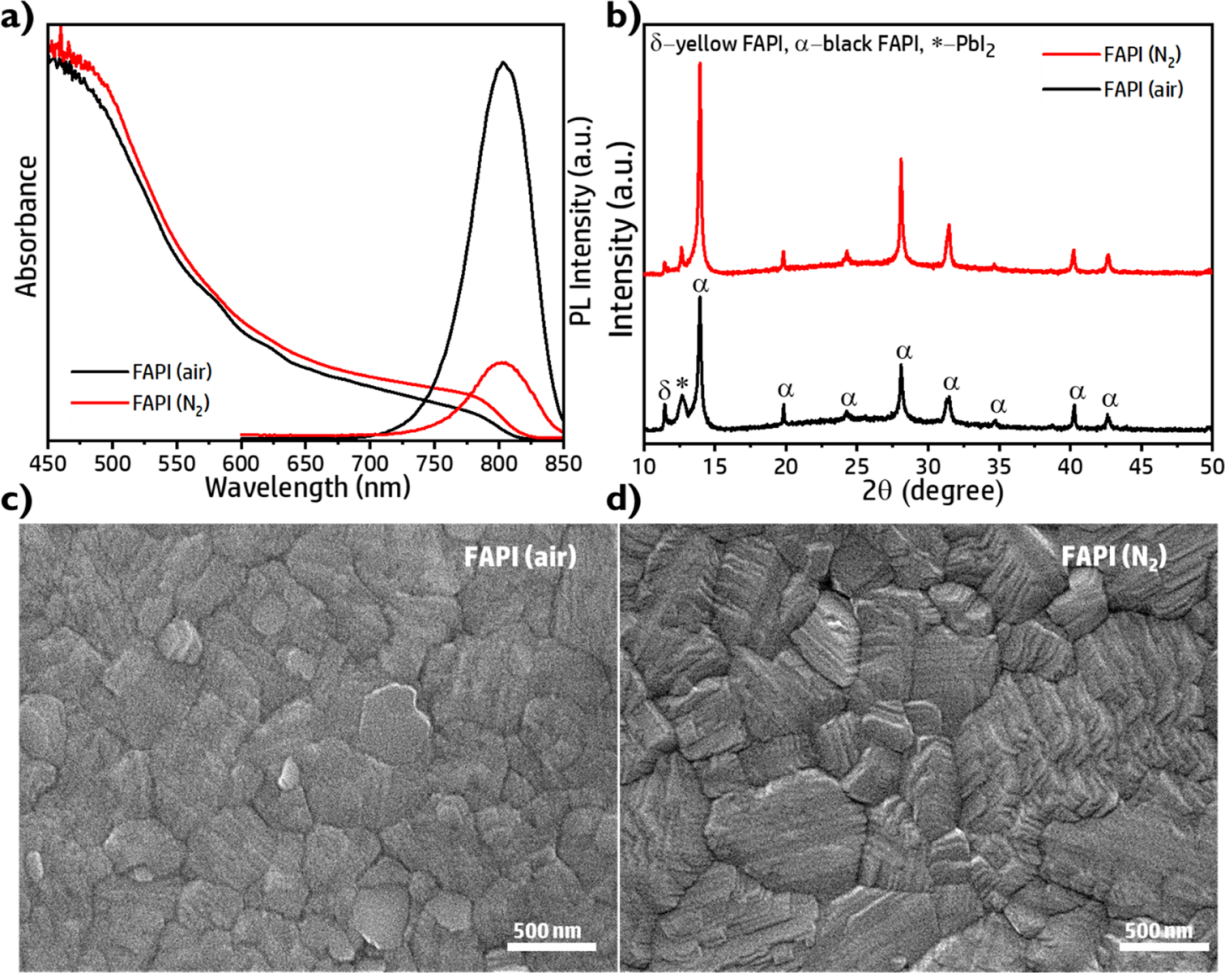
Optical, structural, and morphological features of FAPI perovskite thin films fabricated under air and N_2_ atmospheres. a) UV–vis absorption spectra and steady-state PL spectra. b) XRD) patterns. SEM top-view images of FAPI perovskite thin films fabricated under c) air and d) N_2_ atmospheres, respectively (with the scale bar of 500 nm). The samples for UV–vis absorption, PL, and XRD measurements are fabricated on glass substrates while the samples for SEM and AFM measurements are fabricated on conductive ITO substrates (at 25 °C, RH = 40–60%).

From the structural point of view, in the X-ray diffraction (XRD) patterns we found a small contribution of PbI_2_ (12.70°)^31,45^ and δ-FAPI phase (11.80°),^31,45^ where the latter is more pronounced in the samples prepared under ambient conditions, Figure 1b, causing the shoulders observed in absorption spectra, Figure 1a, at 570 and 620 nm, and the lower light absorption especially in the 750–800 nm region. The diffraction peaks observed at 13.95°, 19.85°, 24.30°, 28.10°, 31.50°, 34.65°, 40.25°, and 42.75° in all spectra are corresponding to (1 0 0), (1 1 0), (1 1 1), (2 0 0), (2 1 0), (2 1 1), (2 2 0), and (2 2 1) of the cubic black phase of FAPI (α-FAPI).^31,45^ The presence of PbI_2_ in the fabricated films has been systematically observed with the solvent and annealing temperature used; see the Experimental Section in the Supporting Information. Moreover, the morphology of the films is slightly different, Figure 1c,d. Scanning Electron Microscopy (SEM) images of the sample prepared under nitrogen have small and intermediate size grains stacked one over another, resulting in higher surface roughness, ∼20 nm, as determined by Atomic Force Microscopy (AFM) when compared to FAPI films from the air, with a surface roughness of ∼10 nm, Figure S2.

Solar cells have been fabricated by adopting a planar n-i-p device structure that consists of indium tin oxide (ITO)/SnO_2_/FAPI perovskite (air/N_2_ or with FAPI-PbS QDs)/2,2′,7,7′-tetrakis(*N*,*N*-di-4-methoxyphenylamino)-9,9′-spirobifluorene (Spiro-MeOTAD)/Au as shown in Figure 2a.^54^ Pure FAPI solar cells have been fabricated following the same procedure under ambient (RH 40–60%) and N_2_ atmosphere, hereafter called FAPI (air) and FAPI (N_2_) samples. In addition, different concentrations of PbS QDs were included as an additive in FAPI layers fabricated under ambient air, hereafter referred to using the PbS QDs concentration as 0.5, 1, 2.5, and 5 mg/mL (air). See the Supporting Information section for details of PbS QDs additive incorporation into FAPI films and optical and structural characterization of the produced films, Figures S3–S7. Different concentrations were investigated as previous works pointed out the performance of the solar cell is influenced by both PbS QDs size and embedded PbS QDs concentration.^28,50^ The resultant perovskite films are methodically characterized, Figure S6. There are no significant differences in terms of structural and optical properties over the concentration of added PbS QDs. However, the presence of FAPI-PbS QDs, which do not affect the emission wavelength and the bandgap value, Figure S6e,f, induced a decrease in the PL emission intensity of perovskite film with respect to reference FAPI (air) film, Figure S6e.^56−58^

**Figure 2:**
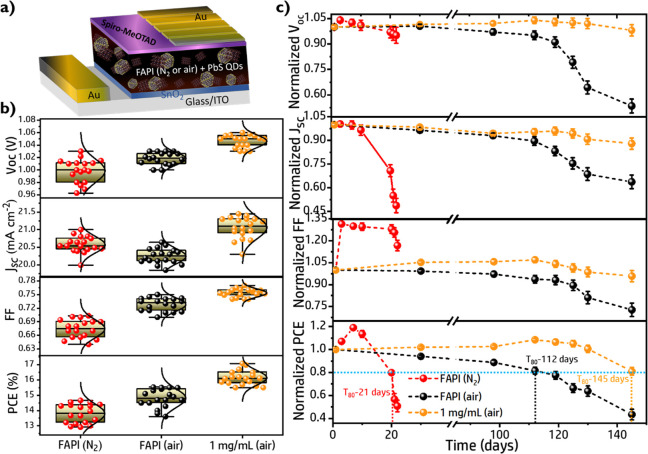
a) Schematics of perovskite solar cell architecture. b) The statistical photovoltaic parameters of FAPI (N_2_), FAPI (air), and FAPI (air) with 1 mg/mL embedded PbS QDs, denoted as 1 mg/mL (air), solar cells. All the PSCs were measured in the ambient air (conditions of 25 °C, ∼RH 60%), and the parameters (*V*_oc_, *J*_sc_, FF, and PCE) were collected from 18 individual devices. c) The long-term stability comparison of the unencapsulated FAPI (N_2_), FAPI (air), and 1 mg/mL (air) devices, the normalized photovoltaic parameters like *V*_oc_, *J*_sc_, FF, and PCE. The data were obtained from the average of 5 different devices, and the devices were stored in ambient air without encapsulation (25 °C, RH 23%, at the dark condition).

Figure S7 shows a comparison of the top-view images of the reference FAPI (air) and FAPI-PbS QDs (air) films by SEM. Both kinds of samples present pinhole free films. FAPI (air) present domains with a size around 450 nm, Figure S7 and Table S3. Amounts 0.5 and 1 mg/mL (air) show larger domains, with the mean size of 550 and 650 nm, respectively, Figure S7 and Table S3. A further increase of QDs concentration reduces FAPI domain size. Diffraction peaks corresponding to the characteristic peak of the hexagonal yellow phase of δ-FAPI and PbI_2_, at 11.80° and 12.70°, respectively, are observed for all the films with and without PbS additive, Figures 1b and S6a,^31,45^ also for films fabricated under a N_2_ atmosphere, Figure S8, as previously reported.^45^ Consequently, the presence of the PbI_2_ peak is not the result of the deposition in ambient condition, with an RH range of ∼40–60%,^59^ but rather it is an effect of the strong and complex interaction between the precursors, FAI and PbI_2_, and solvents, NMP and DMF, in solution, with beneficial repercussion in the solid-state material, as passivation of the grain boundaries previously reported,^17,43,60^ independent of the PbS QDs additives.

PSCs have been fabricated in the air with different amounts of PbS QDs additives, and their performances were systematically characterized, Figures S9 and S10. Figure S11 shows the cross-sectional SEM images of the solar cell devices with and without PbS QDs. The relative thickness of the perovskite layer is found to be similar for reference FAPI (air) and FAPI-PbS QDs (air), ∼300 nm. The current density–voltage (*J*–*V*) curves of champion cells are reported in Figure S12. The optimal FAPI-PbS QDs (air) for the best device performance is found to be for 1 mg/mL (air) additive concentration; all photovoltaic figures of merits such as average and best values of the devices are statistically analyzed in Figure S10 and Table S4.

Photovoltaic parameters of FAPI (N_2_), FAPI (air), and FAPI with the optimized concentration of PbS QDs of 1 mg/mL (air) are compared in Figure 2b. Averaged photovoltaic values and champion cells are summarized in Table S4. FAPI (air) samples present lower average photocurrents, *J*_sc_, than FAPI (N_2_) samples, a fact that could originate from the higher amount of the δ-phase, Figure 1b. However, the higher average fill factor, FF, and open circuit potential, *V*_oc_, eventually increase the average PCE of FAPI (air) with respect to FAPI (N_2_). Interestingly, 1 mg/mL (air) boosts all the photovoltaic parameters in comparison with both FAPI (N_2_) and FAPI (air), obtaining also higher reproducibility with lower dispersion of PCE, Figure 2b. Just slight hysteresis is observed, Figure S12 and Table S5. There is a good agreement between the *J*_sc_ obtained from *J*–*V* curves, Table S4, and integrated incident photon to current efficiency (IPCE), Figure S13. No significant photocurrent can be attributed to PbS QDs absorption. Stabilized PCEs are depicted in Figure S14. The device fabricated in an air atmosphere with 1 mg/mL (air) gave the best PCE of 17.08% with a *V*_oc_ of 1.06 V, a *J*_sc_ of 21.43 mA cm^–2^, and a FF of 0.77, Table S4.

The enhancement in performance of 1 mg/mL (air) devices could be related to the morphological changes observed in films with an increase of the crystalline domains when a low concentration of FAPI-PbS QDs is used, Figure S7, which lead to fewer defects associated with lower recombination, and in turn with the decrease of the charge transport and extraction.^61,62^ Also note that a negligible hysteresis is observed in the case of 1 mg/mL (air) in comparison with FAPI (air) and FAPI (N_2_), which also points in the same direction.^63^

In order to gain more understanding of the charge recombination dynamics and trap density of the optimized perovskite material prepared under ambient conditions, we studied the time-resolved photoluminescence (TRPL), space-charge-limited-current (SCLC), and electrochemical impedance spectroscopy (EIS) measurements. EIS shows the slightly higher recombination resistance^64^ for 1 mg/mL (air) films, Figure S15. The TRPL measurement is performed on bare glass, and all the PL curves, Figure S16a, are fitted with a double exponential decay function, as shown in Table S6, to elucidate the fast and slower decay kinetics. It is observed that the charge carriers are relatively long-lasting in the reference FAPI (air) films than 1 mg/mL (air) films. In this context it is worth pointing out that the effect of PbS addition in the steady-state PL and TRPL behavior is the result of two concomitant and opposite mechanisms: (i) the increase of FAPI domain size due to the seed effect for crystal growth of PbS QDs additives and (ii) the PbS QDs acting as nonradiative recombination center.^56,57^ The space charge-limited current (SCLC) is measured to quantitatively evaluate the trap density (*n_trap_*) in the FAPI (air) and 1 mg/mL (air) perovskite films. Figure S16b, shows the dark current–voltage (*I*–*V*) curves of electron-only devices with the configuration of ITO/SnO_2_/FAPI (air) or 1 mg/mL (air))/PCBM/Au. The reference FAPI (air) device shows a higher trap density, *n_trap_*, of 3.8 × 10^16^ cm^–3^, which is 1.8 times larger than the *n_trap_* of 2.1 × 10^16^ cm^–3^ of the 1 mg/mL (air) device which agrees with the reduced *V*_oc_ and FF in the reference device. The lower *n_trap_* in the 1 mg/mL (air) device is attributed to the improved film quality, causing the improvement of *V*_oc_ values and the presence of alleviated *J*–*V* hysteresis in the devices.^61,62,65^

Nevertheless, beyond the PCE enhancement observed for PSCs fabricated in air, especially when PbS QDs are used as additives, the most dramatic change in performance comparing air and N_2_ fabricated solar cells is the improvement of the long stability of the former, Figure 2c. All PSCs were measured in the same air conditions (25 °C, RH 60%) during the time, and it was found that the devices prepared under nitrogen were more vulnerable to the atmosphere, Figure 2c. For FAPI (N_2_) after an initial improvement of the *V*_oc_ and FF, likely due to the ambient effect,^18^ all the parameters decreased very quickly, until reaching a very low PCE of 4.3% after less than 1 month. On the contrary, the performance of unencapsulated FAPI (air) exhibited an outstanding stability with a very low performance decrease during the first 100 days. After that, the degradation speeded up reaching *T*_80_ after 112 days. The addition of PbS can increase further the long-term stability of sample fabricated in air *T*_80_ = 145 days for 1 mg/mL (air) devices, Figure 2c. It is important to highlight that the PbS additive increases the *T*_80_ with respect to the FAPI (air) samples for all the PbS QDs concentrations studied in this work, Figure S17. In fact, after 145 days samples with a higher concentration of PbS QDs retain better performances; 5 mg/mL (air) samples presented 90% of their initial PCE even up to 145 days after their fabrication, Figure S17d. These results point to a beneficial stability role of PbS QDs additive beyond the increase of PCE, likely associated with the chemi-structural stabilization of α-FAPI phase induced by PbS QDs incorporation.^28^ Moreover, the addition of PbS QDs also increases significantly the stability under continuous illumination, Figure S18.

The XRD patterns of the 30 days aged unencapsulated complete devices show important differences among the different kinds of analyzed samples, Figure S19. Enhanced formation of impurity PbI_2_ peaks is observed for the reference FAPI (air) devices when compared to the 1 mg/mL (air) device, but no increase of the weight of the FAPI δ-phase is observed. In contrast, the presence of δ-phase is dominant for the sample fabricated in N_2_. The complete aging data for the reference FAPI (air) and FAPI-PbS QDs (air) materials at different aging times including XRD patterns, film photographs, SEM images, AFM images, and light absorption are reported in Figures S20–25, respectively, and with the direct comparison between FAPI (air) and 1 mg/mL (air) in Figure S26. Complete devices present higher stability than thin films pointing to the protective effect of selective contact layers. Note, in addition, the clear beneficial effect of PbS additives from the stability point of view, as stability is enhanced for all the analyzed PbS QDs concentrations compared with FAPI (air), but also the improvement in stability observed for FAPI (air) with respect to FAPI (N_2_).

While the mechanism that enhances the long-term stability of FAPI based PSCs has been previously determined,^28^ the effect of air fabrication remains unclear. To clarify this important point, systematic Raman and high-energy-resolution photoemission spectra (XPS) studies have been performed. While the morphology and XRD of FAPI (air) and FAPI (N_2_) films are very similar, Figure 1b−d, the comparative Raman spectra analysis of perovskite thin films of FAPI, prepared under air and N_2_, present interesting differences, Figure 3.

**Figure 3:**
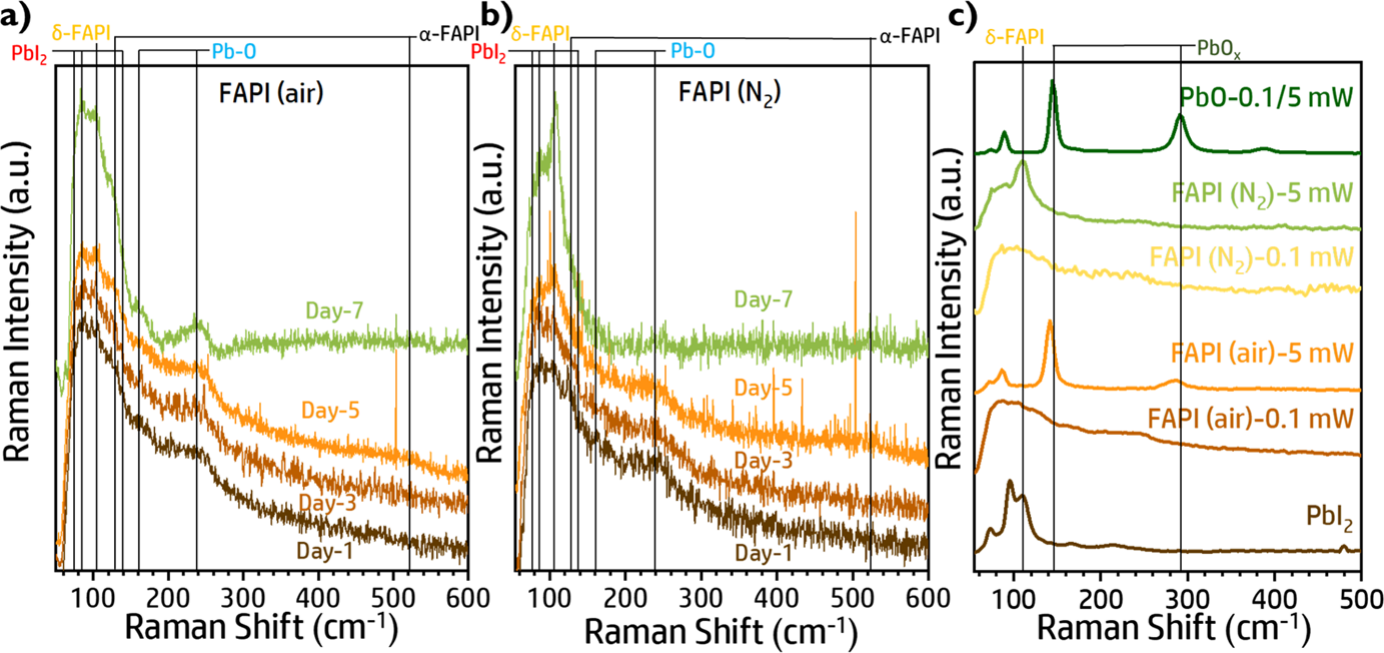
Raman spectra of perovskite films on the glass substrate, measured at ambient air by excitation with a 532 nm laser with a low power intensity of 0.1 mW. The data have been normalized, and the vertical lines indicate spectral mode assignment. Raman spectra of aged films (day 1, 3, 5, and 7) for a) FAPI (air) and b) FAPI (N_2_). c) Raman spectra comparison of FAPI (air) and FAPI (N_2_) films measured at 0.1 and 5 mW power intensity, compared with the Raman spectra of PbI_2_ and PbO crystal powder.

Figure 3a,b shows the Raman spectra of aged FAPI (air) and FAPI (N_2_) perovskite thin films, respectively, using an excitation wavelength of 532 nm and low excitation power intensity, 0.1 mW. The Raman spectra of fresh FAPI (air) and FAPI (N_2_) films possess relatively similar Raman vibrational modes. As indicated in Figure 3a,b, a low-energy shoulder below 100 cm^–1^ and extending to 65 cm^–1^ is from the PbI_2_ phonon modes. A small low-energy mode at 107 cm^–1^ denotes the presence of δ-FAPI and another much broader Raman band positioned at 135 and 520 cm^–1^, in-plane bending of FA cations, are from the presence of α-FAPI.^66,67^ Moreover, the presence of prominent Raman modes at 163 and 238 cm^–1^, respectively, are expected from the oxygen-related nature at the surface, i.e., oxygen ingression into the perovskite film and its intensities being relatively higher in FAPI (air) films,^66,67^ pointing to air preparation increasing the amount of Pb–O bonds.

Over time, the samples prepared in different atmospheres present a clearly different evolution. Raman modes associated with PbI_2_ and δ-FAPI, below 100 cm^–1^ and at 107 cm^–1^, respectively, exhibit no relative evolution during 1 week aging for FAPI (air), Figure 3a, while a more pronounced clear increase for δ-FAPI modes is observed for FAPI (N_2_), Figures 3b and S27. In contrast, Raman modes at 163 and 238 cm^–1^, associated with Pb–O bonds, are clearly defined for FAPI (air) after 7 days, while no evolution, even an attenuation, of these modes is observed for FAPI (N_2_), Figures 3b and S27.

Moreover, film degradation can be also forced by increasing the laser power of Raman analysis.^67^ The spectra of FAPI (air) are changed at higher power intensity (5 mW) and the observed spectra can be related to lead oxide products (e.g., PbO, PbO_2_, etc.), Figure 3c and Figure S28. Raman modes at 140 cm^–1^ and two phonon modes at 281 cm^–1^ are indicative of the formation of PbO_*x*_ products and it shows the characteristics of the Pb–O bonds in the perovskite films.^66,67^ Note that no Raman peak associated with the δ-FAPI modes at 107 cm^–1^ is observed for the high-laser-power degraded FAPI (air) sample, Figure 3c. On the contrary, films prepared under N_2_ intentionally degraded using high laser power do not show any signature of the formation of PbO_*x*_ products; in contrast, an increase of δ-FAPI mode intensity with aging is observed, Figure 3c.

From the morphological point of view, changes in the evolution of FAPI (air) and FAPI (N_2_) films are also clearly seen with bare eyes after 5–7 days of aging. While the FAPI (air) films retain their black color characteristic of the α-phase, Figure S24, FAPI (N_2_) films become progressively yellow, Figures S24 and S27. In 5–7 days of aged FAPI (N_2_) films, two different areas were observed, area 1 and area 2, and the Raman spectra acquired indicate that bright spots, i.e., area 2, correspond to δ-FAPI phase regions,^66^Figure S27a–c. Interestingly no peaks associated with Pb–O bonds are observed in these regions while it is observed in the area 1 region with the α-FAPI phase. The aging time increases the size of the δ-FAPI phase regions, and no Raman modes associated with Pb–O bonds are observed in this region, Figure S27.

Based on Raman spectra studies, we further recorded the XPS of FAPI (air and N_2_) films to probe the chemical changes occurring over the aging period. Figure 4 shows the Pb 4f and I 3d core-level spectra measured in FAPI (air) films (panels a and b, respectively) and in FAPI (N_2_) films (panels c and d, respectively). The Pb 4f and I 3d spectra recorded in the fresh films appear to be composed of single spin–orbit doublets located at similar binding energies in FAPI (air) and (N_2_) films. In these fresh samples, the Pb 4f_7/2_ component lies at 138.5 eV (the Pb 4f spin–orbit splitting results 4.9 eV) and the I 3d_5/2_ component lies at 619.3 eV (the I 3d spin–orbit splitting results 11.5 eV), which can be attributed to the major presence of Pb–I bonds in these perovskites.^68,69^

**Figure 4:**
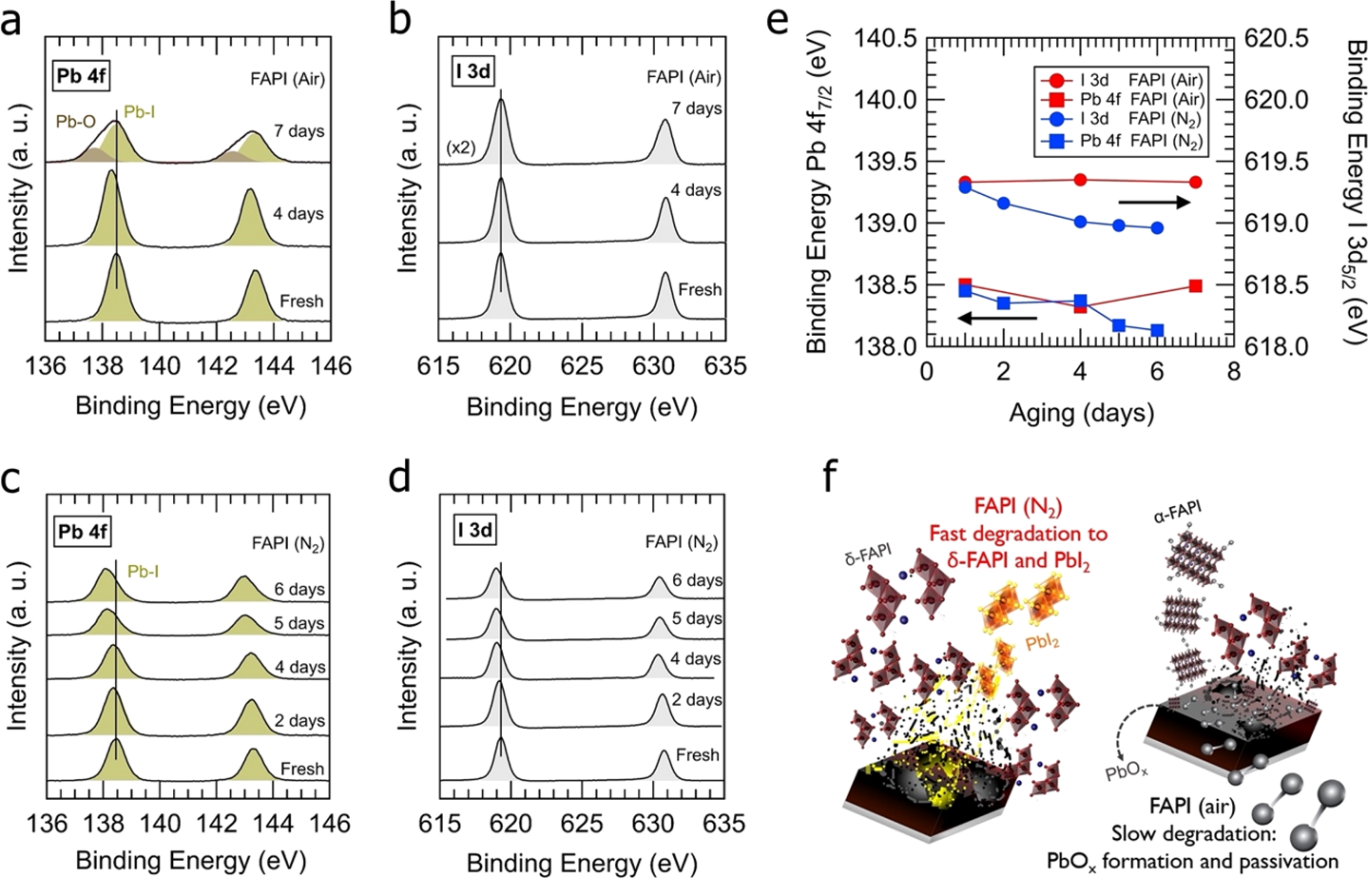
High-energy-resolution XPS spectra of Pb 4f and I 3d measured in (a and b) FAPI (air) and (c and d) FAPI (N_2_) films with a different aging degrees, which range between fresh and 7 days aged samples. The aging is indicated on each curve. Vertical solid lines in (a) and (c) mark the energy position of the Pb 4f_7/2_ component acquired in the fresh samples. e) Binding energy dependence on aging of the Pb 4f_7/2_ and I 3d_5/2_ components attributed to the Pb–I bonds existing in FAPI (air) and FAPI (N_2_) films, as extracted from Gaussian fitting of the spectra shown in (a–d). f) Schematic representation that shows the degradation pathways of FAPI (air) and FAPI (N_2_) films under ambient aging conditions.

Comparing samples fabricated under ambient air or N_2_ shows a clear difference in the aging evolution of these two kinds of samples. In the case of FAPI (air) samples, aging favors the development of an additional Pb 4f doublet at lower energies (with a Pb 4f_7/2_ component located at 137.7 eV). The appearance of this new Pb 4f component, in 7-days aged films, is accompanied by a decrease of the XPS signal intensity of the corresponding I 3d core level (Figure 4b). These facts suggest a progressive formation of lead(II) oxide (PbO).^68^ In addition, peak position associated with Pb–I bond experience no shift with aging, Figure 4a,e. No displacement is observed for the I 3d peaks either, Figure 4b,e. Moreover, we observed no significant peak attributable to Pb^0^ (expected at 136.9 eV) and higher binding energy broadening that may point out to the formation of lead carbonate (PbCO_3_, at a binding energy of 139.3 eV).^68,69^ In the case of aged FAPI (N_2_) films, however, no traces from additional Pb 4f components have been observed, Figure 4c. In addition to this, aging effects seem not to reduce the presence of I in these films, Figure 4d. Instead, the Pb 4f and I 3d core levels attributed to the Pb–I bond appear to downshift by 0.3 eV in 7 days of aging, Figure 4e. The behavior of the core level peaks of the FAPI (N_2_) films observed over aging can be associated with a phase conversion from α-FAPI to δ-FAPI without any presence of intermediate products (such as PbO, PbCO_3_, etc.).^68^ This transition implies a change in the Pb and I bonding nature as neighbor octahedra in δ-FAPI are no longer sharing iodine corners as in α-FAPI. Though the negligible shift for the Pb 4f and I 3d binding energy of FAPI (air), Figure 4e, and the clear observation of Pb–O bonds points to the α-FAPI phase mediated by PbO products on the films that block the formation and propagation of δ-FAPI. In addition, as shown in Figure S29, the O 1s spectra of the FAPI (air and N_2_) samples, clearly shows the evolution of more oxygen content in the aged FAPI (air) over the FAPI (N_2_) samples which exhibited only a trace amount of the oxygen contents.

Figure 4f shows the schematic representation of the degradation pathways of FAPI (air) and FAPI (N_2_) films under ambient aging conditions. In correlation with Raman, XPS, and morphological analysis, results indicate that the presence of Pb–O bonds blocks the expansion of the δ-FAPI phase, stabilizing the FAPI black phase. These findings suggested that the fabrication at ambient conditions intrinsically form Pb–O bonds, likely PbO_*x*_ species which would improve the air stability. Degradation produces an increase of the oxidized species, Figure 3c and Figure 4a, rather than δ-FAPI phase formation. In this line, an increase of stability in Sn–Ge perovskite solar cells due to the presence of oxide species has been recently reported.^70^ On the contrary when the perovskite film is prepared under nitrogen, without passing through the PbO_*x*_ species, the exposure to air provokes the degradation with the formation of PbI_2_ and δ-phase, Figure S27 and Figure 4f. Thus, the specific preparation conditions with the NMP and the ambient conditions enable the formation of Pb–O bonds to obtain more stable pure FAPI solar cells producing a dramatic increase in device stability.

The main scope of this work is the systematic investigation of stability enhancement of FAPI-based PSCs. In order to perform this study, we considered that the most valuable results could be obtained in the harsher situation dealing with pure FAPI PSCs, with the potential of being extrapolated to other systems. To this extent, it is also interesting to analyze the effect of other additives. The most extended additive employed for the formation of high-performance FAPI-based PSCs is MACl.^29,30,41,42^ MACl helps in the layer crystallization with an important impact in the final PCE. The use of MACl can produce the final incorporation of MA into FAPI films in percentages lower than 10%.^29,41^ This incorporation can slightly blue-shift the material bandgap but also helps in the FAPI phase stability. The effect of MACl, 20 mol %, in FAPI-based PSCs fabricated in ambient conditions with RH = 40–60% has been investigated, as in the previous report, the use of this additive significantly increases the cell performance, Figure 5.

**Figure 5:**
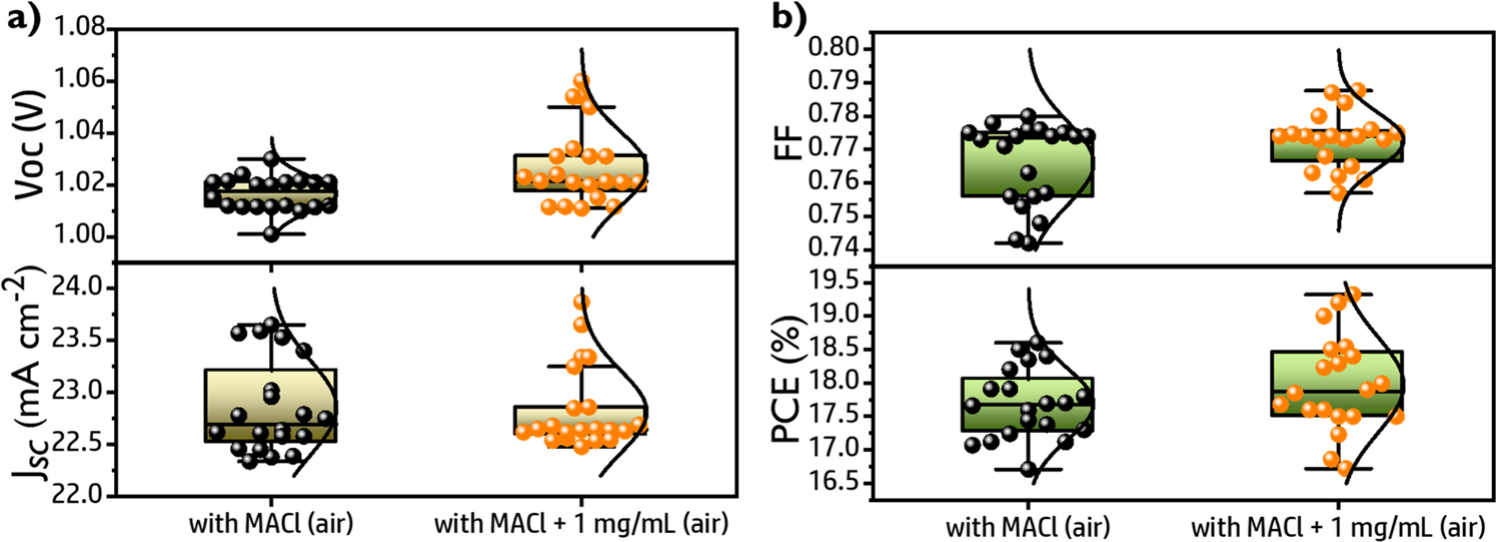
Statistical photovoltaic parameters obtained from 20 devices for reference MACl (air) and MACl + 1 mg/mL (air): a) *V*_oc_ and *J*_sc_; (b) FF and PCE.

When MACl additive is used, the incorporation of PbS QDs still increasing the average PCE due to the increase of all photovoltaic parameters *V*_oc_, *J*_sc_, and FF, Figure 5 and Table S7. The champion performance for pure FAPI PSCs containing 1 mg/mL (air) of PbS QDs is boosted from 17.08% to 19.38% by the addition of the MACl additive. Note that this performance has been obtained in devices fabricated in ambient conditions with moderate/high RH of 40–60%, significantly higher than the reported previously.^29,41^ In fact we have observed PCE as high as 19.6% for MACl-FAPI (air) PSCs fabricated with RH ∼30%, Figure S30. Incorporation of MACl additive especially increases *J*_sc_ and FF, Figure S31 and Table S7. Samples with MACl also present negligible hysteresis, Figure S31a and Table S8. The increase of *J*_sc_ has been corroborated by the IPCE measurements, Figure S31b. It is important to highlight that unencapsulated devices prepared with MACl additive increase their performances 3 days after fabrication and maintain 100% of the original performance for at least 53 days, Figure S32.

In summary, in this study, the effect of ambient fabrication in improving photovoltaic performance and longevity of ambient air fabricated pure FAPI based perovskite solar cells is demonstrated. It has been observed that films prepared in the air with NMP additive take benefits from the presence of Pb–O bonds, which block the propagation of δ-FAPI phase. This effect boosts pure FAPI device stability *T*_80_ from 21 days to 112 comparing devices prepared in N_2_ and air atmospheres, respectively. Moreover, the addition of PbS QDs for samples prepared in ambient conditions with RH = 40–60% increases both PCE and long-term stability. Devices with optimized PbS QDs achieved a photoconversion efficiency of 17.1% and showed an improved perovskite structural stability, observing unprecedented values, for unencapsulated FAPI films, of *T*_80_ of 145 days, of storage under an ambient atmosphere with an RH of 23%. Notably, an increase of stability is observed for all the PbS QDs concentrations (0.5–5 mg/mL) studied in this work, pointing to a chemi-structural stabilization of α-FAPI phase beyond the increase of solar cell performance. Photoconversion efficiency can be further improved by the use of MACl additive obtaining a champion PCE of 19.4% for PSCs with 1 mg/mL PbS QDs for samples prepared under ambient air conditions with RH = 40–60%. It is shown that both MACl and PbS QDs present an additive effect observing the higher performance in PSCs with both. This work stresses the potential for significant long-term stability improvement in FAPI-based PSCs, the ones with the highest theoretical performance, like present the bandgap closer to the ideal for maximum photovoltaic performance as determined by the Schockley–Queisser limit. Moreover, the δ-phase blocking effect of the Pb–O bonds opens the way for the application of this strategy to other polymorphic perovskite materials not stable in the air, e.g., perovskites with stable yellow phase at room temperature or lead-free perovskite, for environmental stable optoelectronic devices.

## Supporting Information Available

Experimental section, XRD patterns, UV–vis spectra, PL spectra, Raman spectra, XPS spectra, photographs of the perovskite thin films, SEM images, AFM images, TRPL data, current–voltage scan (*J*–*V*) curves, IPCE spectra, device statistics data (such as *V*_oc_, *J*_sc_, FF, and PCE), stabilized photocurrent density at maximum power point vs time, recombination resistance versus applied voltage; tables of XRD peak area comparison, bandgap values, statistical parameters, data for PL curves, photovoltaic performance characteristics, (PDF)
