# Supplementary material for: Boosting Long-Term Stability of Pure Formamidinium Perovskite Solar Cells by Ambient Air Additive Assisted Fabrication
Source: ACS Energy Lett. 2021 Sep 13;6(10):3511–21. doi: 10.1021/acsenergylett.1c01311 (PMC8506569; doi:10.1021/acsenergylett.1c01311)
Supplement: Supplementary file 1 — nz1c01311_si_001.pdf [file nz1c01311_si_001.pdf]

Supporting Information

Boosting Long Term Stability of Pure Formamidinium Perovskite Solar Cells by Ambient Air Additive Assisted Fabrication

K. M. Muhammed Salim,[1] Sofia Masi,[1] Andrés Fabián Gualdrón-Reyes,[1] Rafael S. Sánchez,[1] Eva M. Barea, [1] Marie Krecmarová,[2] Juan F. Sánchez-Royo,[2,3] and Iván Mora-Seró[1*]*

[1] Institute of Advanced Materials (INAM), University Jaume I, Avenida de Vicent Sos Baynat, s/n, 12071 Castelló de la Plana, Castellón, Spain

[2] Institute of Materials Science (ICMUV), University of Valencia, c/ Catedrático José Beltrán, 2, 46980 Paterna, Valencia, Spain

[3] MATINÉE: CSIC Associated Unit (ICMM-ICMUV of the University of Valencia), Universidad de Valencia, Valencia, Spain

Corresponding authors emails: masi@uji.es; sero@uji.es

1. Experimental Section

Materials and methods

All the materials are reagent grade and used without further purification. Formamidinium iodide (FAI, 98%, from GreatCell solar), Methylammonium chloride (MACl, 98%, from GreatCell solar), Lead iodide (PbI_2, > 98%, from TCI). N,N-dimethylformamide (DMF, Anhydrous, 99.9%, Sigma Aldrich), N-Methyl-2-pyrrolidone (NMP, Anhydrous, 99.9%, Sigma Aldrich), Diethyl ether (Anhydrous, >99.8%), Chlorobenzene (CB, Anhydrous, 99.8%), Acetonitrile (MeCN, Anhydrous, 99.8%), 4-tert-butylpiridine (*t*BP, 96%), Zinc powder (99.995%), Lithium bis(trifluoromethylsulfonyl)imide (Li-TFSI, 99.95%), and Hydrochloric acid (HCl, 37%) from Sigma Aldrich. Ethanol and Acetone from PanReac, 2-isopropanol (99.7% from Carlo Erba). 2,20,7,70-tetrakis[N,N-di(4-methoxyphenyl)amino]-9,90-spirobifluorene (Spiro-MeOTAD 99% from Feiming chemical limited), Toluene (Anhydrous

99.8%) from VWR, Octane and SnO_2 colloid precursor from Alfa Aesar (15% in H_2O colloidal dispersion). Indium tin oxide (ITO) coated glass substrates (Pilkington TEC-15, ~ 15 Ω sq^{-1}).

Ligand exchange of PbS QDs

The good-quality PbS QDs are synthesized according to the reported procedure.[1] In a typical experiment, a 0.25 M of FAPI solution (in DMF) and 10 mg/mL of PbS QDs solution (in octane) are mixed in an equal volume ratio (v/v of 1:1) and stirred for 30 min at 30°C. During the reaction, the PbS QDs are transferred from the nonpolar-octane phase to the polar-DMF phase. Subsequently, the DMF phase is washed three times with octane to remove the excess organic ligands. Then, the ligand exchanged PbS QDs (named as FAPI-PbS QDs) are precipitated by the addition of toluene. Finally, the FAPI-PbS QDs are dried under vacuum for 2-3 hours.

Device fabrication

The Indium tin oxide (ITO) coated glass was etched with Zn powder and 6 M hydrochloric acid and followed by 15 minutes of ultrasonication cleaning with decon soap solution, deionized water, acetone, and isopropanol. The ITO substrates were dried with dry air and performed UV-Ozone treatment before the electron transport layer (ETL) deposition. The SnO_2 ETL was deposited by spin-coating of 2.65 wt% of colloidal SnO_2 in deionized water at 3,000 rpm for 40 seconds, and substrates were annealed at 150 °C for 30 minutes. The $FAPbI_3$ perovskite solution (hereafter named as FAPI) was prepared by dissolving 461 mg of PbI_2 (1 mmol) and 172 mg of FAI (1 mmol), 96.30 µL of N-Methyl-2-pyrrolidone (NMP, 1 mmol) in N,N-dimethylformamide (DMF, 590.71 µL) and the solution was heated at 60°C, (for the MACl additive based solution, in addition to the FAI (1 mmol), PbI_2 (1 mmol), 13.50 mg of MACl (0.2 mmol) was dissolved in the same DMF:NMP solvent system). The dried FAPI-PbS

QDs were dispersed in 400 μL of FAPI solution to get a 25 mg/mL of stock solution. The stock solution of FAPI-PbS QDs was further diluted for the preparation of 0.5, 1, 2.5, and 5 mg/mL solutions. Before the perovskite deposition, SnO_2 ETL substrates were treated with UV-Ozone for 20 minutes. The perovskite solution (with and without FAPI-PbS QDs) was spin-coated at 5000 rpm for 17 seconds and 400 μL of diethyl ether solution was dropped on the spinning substrate at 9th second. The substrate was then annealed at 100°C for 1 minute and 165°C for 10 minutes.[2, 3] Subsequently, the Spiro-MeOTAD solution was deposited by spin coating at 4000 rpm for 35 s. The Spiro-MeOTAD solutions were prepared by dissolving (72.3 mg/mL in 1 mL chlorobenzene) and followed by the addition of tert-butyl pyridine (t-BP, 28.8 μL), Lithium bis(trifluoromethylsulfonyl) imide (Li-TFSI, 17.5 μL). Finally, 80 nm of the gold counter electrode was deposited using a thermal evaporator. Note that, the perovskite thin film and Spiro-MeOTAD deposition were carried out at ambient atmosphere conditions (with a relative humidity of ~40-60%).

Structural characterization

X-ray diffraction (XRD): The crystallographic information of the films were analyzed by an X-ray diffractometer (D8 Advance, Bruker AXS) (Cu Kα, the wavelength of l = 1.5406 Å) within the range of 10–55° with a step size of 0.02°. The X-ray diffraction patterns were recorded from films deposited on top of the glass substrate.

Scanning Electron Microscope (SEM): The topographical and cross-sectional images were recorded by field emission scanning electron microscope (FEG-SEM) JEOL 3100F) operated at 5 kV. The SEM was recorded from films deposited on top of the ITO substrate.

Optoelectronic characterization

UV-Vis Spectra: The absorption spectra were obtained using a UV/Vis absorption spectrophotometer (Varian, Cary 300). The UV-Vis spectra were recorded from films deposited on top of the glass substrate.

Photoluminescence Spectra (Visible and infrared), Time-Resolved Photoluminescence (TRPL) Decay profile

The emission measurement (PL spectra) and time-resolved PL (TRPL) decay were collected by Horiba Fluorolog. The photoluminescence spectra (visible and infrared) of perovskite thin films were recorded by exciting the samples at 532 nm. The PL (visible) and PL decay were recorded from films deposited on top of the glass substrate. The PL (infrared) was measured for the PbS QDs and FAPI-PbS QDs from the solution.

Incident-photon-to current conversion efficiency (IPCE): Measured using a QEPVSI-b Oriel measurement system and measured in DC mode.

Electrochemical Impedance Measurements (EIS) were performed by applying 20 mV of a voltage perturbation at different frequencies from 1MHz to 0.1Hz in a PGSTAT-30 from Autolab and under 1 sun illumination at different applied potential, from 0 to 1.2 volts. The recombination resistance was calculated by using the equivalent circuit previously reported.[4]

Solar cell characterization: The current-voltage (J-V) curves are measured using a Keithley 2612 source meter under AM 1.5 G (1000 Wm^{-2}) provided by a Solar Simulator Abet, Xenon short-arc lamp Ushio 150 watts, in the air at a temperature around 25°C and without encapsulation. Each curve is generated using 123 data points from a starting potential of 1.2 V to a final potential of -0.02 V (reverse scan; vice versa for the forward scan) using a scan rate of 10 mVs^{-1}. The active area of the cell is 0.121 cm^2. The photovoltaic performance of the aged devices is measured without any encapsulation, at ambient atmosphere, and under 1 sun condition.

Raman spectroscopy: The micro-Raman spectra were measured by WiTec apyron equipment at 532 nm excitation wavelength with an EMCCD detector. The laser power intensity used was 0.1 mW and 5 mW. The Raman experiments were conducted in ambient air conditions with a relative humidity of 55%.

Atomic Force Microscopy (AFM): The morphology and surface roughness of the perovskite film were measured by using AFM (Concept Scientific Instrument) in resiscope mode. The scanning area was 10x10 μm^2.

X-ray photoemission (XPS) measurements were performed in a SPECS GmbH system (base pressure 1.0×10^{-10} mbar) equipped with a PHOIBOS 150 2D-CMOS hemispherical analyzer. Photoelectrons were excited with the Al-K_a line (1486.7 eV) of a monochromatic x-ray source μ-FOCUS 500 (SPECS GmbH). Measurements were taken at room temperature with a pass-energy of 20 eV.

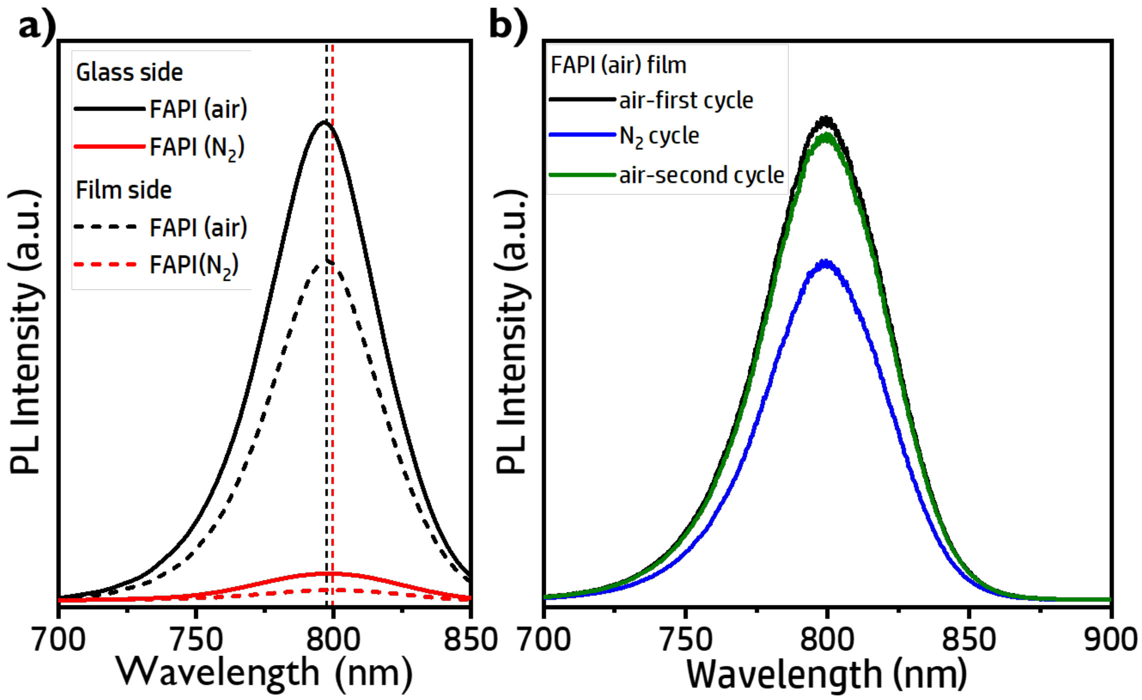

Figure S1. The steady-state photoluminescence (PL) spectra of FAPI (air) and FAPI (N_2) perovskite thin films; a) from the front and backside, b) measurements at ambient air and N_2 purging cycle.

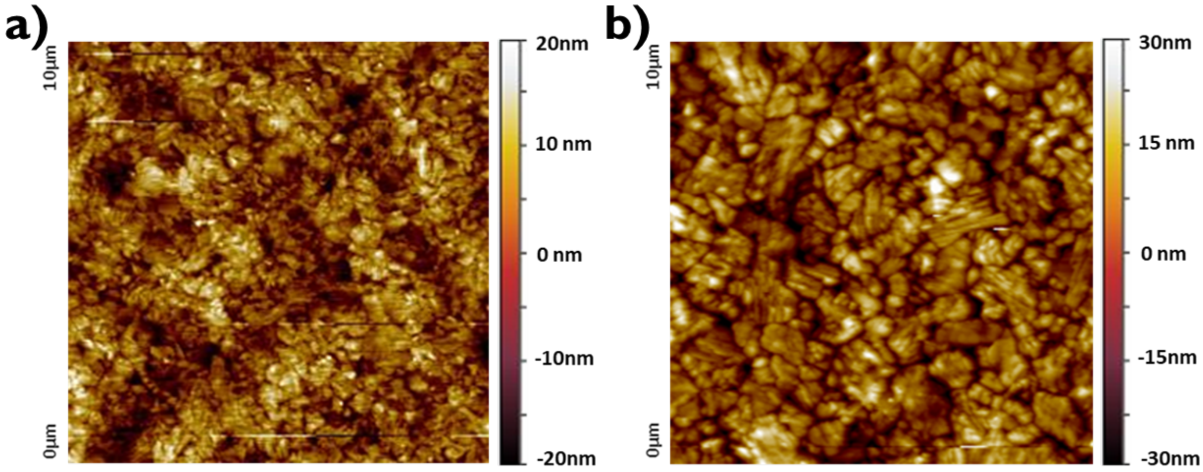

Figure S2. Atomic Force Microscopy (AFM) topography of FAPI perovskite thin films fabricated under a) air and b) N_2 atmosphere, respectively (the scanned area is 10 x 10 µm^2).

PbS QDs additive optimization

We have shown that the addition of PbS QDs causes an increase in the long-term stability of FAPI[5] and FACsPbI[6] solar cells prepared in an inert atmosphere. Here, the effect of PbS QDs under ambient conditions has been analyzed. PbS QDs have been added in the fabrication for the analysis of their effect, hereafter named as FAPI-PbS QDs samples. In this case, prior to mixing the PbS QDs with FAPI precursor solution for the fabrication of perovskite thin films, we have performed the ligand exchange procedures as reported earlier.[5] As shown in Figure S3a, the schematics of phase transfer reaction of the PbS QDs with the long organic chain of oleic acid ligands (OA-PbS QDs) in octane with the FAPI perovskite precursor as short polar ligands in DMF solution. Subsequently, the ligand exchanged products (hereafter named as FAPI-PbS QDs) are transformed into the polar phase, and further precipitated. The newly formed FAPI-PbS QDs are dissolved in FAPI solution for the fabrication of perovskite thin films for the characterization and device applications (Figure S3b). Figure S4a and b show the infrared absorbance and photoluminescent spectra of the OA-PbS QDs and FAPI-PbS QDs. The ligand exchanged FAPI-PbS QDs shows a redshift in photoluminescence,[7] resulting from the reduction of the degree of quantum confinement with the formation of ligand shell.[7] Moreover, there is a very slight blue shift in PL peak for the

FAPI-PbS QDs dissolved in FAPI solution when compared to the solution from pure DMF:NMP solvent, this may be originated from the interaction of the FAPI shell of FAPI-PbS QDs with the perovskite precursor ions.[8, 9]

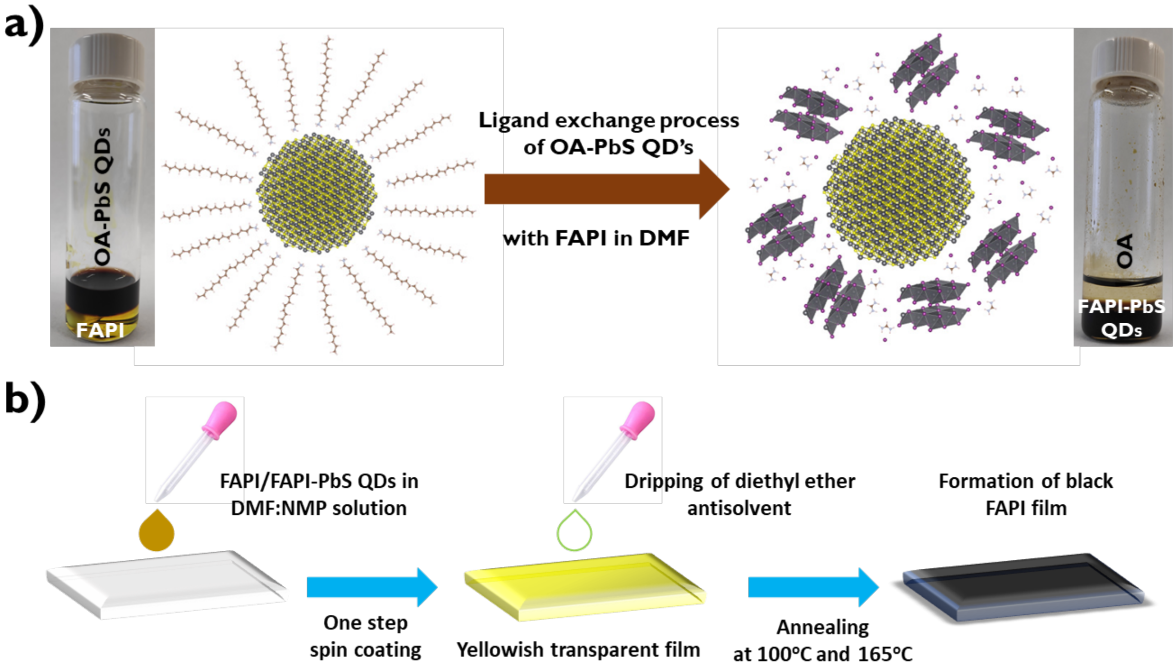

Figure S3. a) Schematic illustration showing the ligand exchange process of OA-PbS QDs with the FAPI solution. The OA-PbS QDs is in the octane solution (10 mg/mL) and the concentration of FAPI is 0.25 M in DMF. OA- refers to oleic acid and FAPI is for $FAPbI_3$. b) Schematic illustration showing the fabrication of FAPI perovskite film with or without FAPI-PbS QDs by spin coating method at ambient atmosphere.

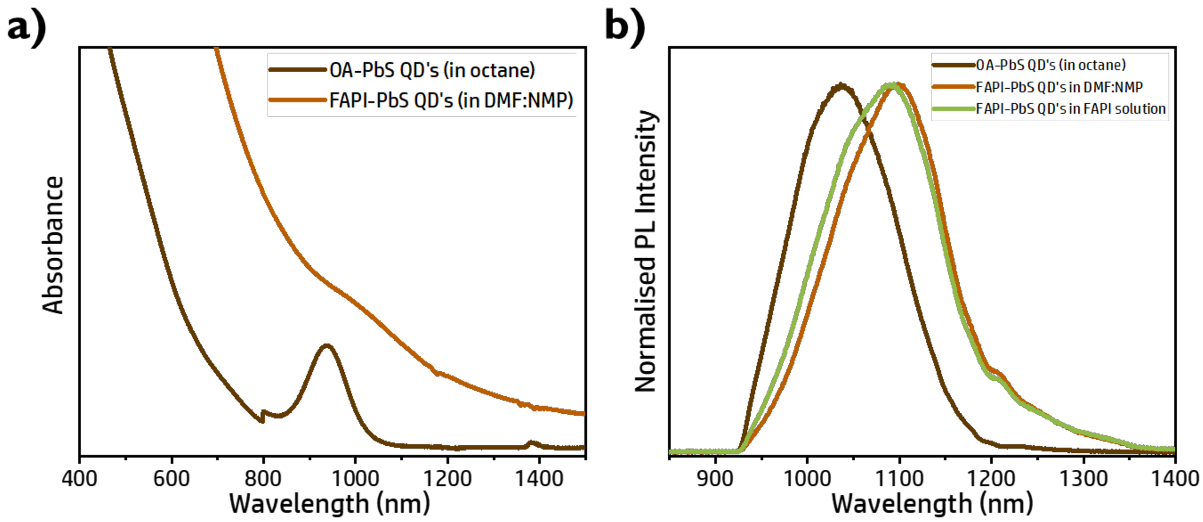

Figure S4. a) The infrared absorbance spectra of OA-PbS QDs before and after ligand exchange process with FAPI solution. The absorbance of PbS-OA is recorded from octane solution, while the ligand exchanged product FAPI-PbS QDs is from DMF:NMP solution. b) The steady-state infrared photoluminescence spectra of PbS QDs, before ligand exchange (mentioned as OA-PbS QDs in octane) then after the ligand exchange with FAPI solution (mentioned as FAPI-PbS QDs in DMF:NMP solution), also dispersed in FAPI perovskite precursor solution (mentioned as FAPI-PbS QDs in FAPI solution).

As shown in the schematics of Figure S3b and reported earlier,[2] the as-spun perovskite films with FAPI-PbS QDs show flat and homogeneous aspects of film surface as well as the reference FAPI (air). Figure S5 shows the comparative X-ray diffraction (XRD) patterns of the just spun (not annealed) perovskite thin films of FAPI (air) and 1 mg/mL FAPI-PbS QDs which formed a non-perovskite phase of FAPI (δ-FAPI), which indicates the absence of other intermediate phase formation with the FAPI-PbS QDs. Despite the high formation temperature of 165 °C to obtain the perovskite black phase (see Experimental section),[2] we observed that the perovskite films annealed only at 135 °C show a more intense peak at 13.95° addressed to the black phase of FAPI (α-FAPI) for the samples with FAPI-PbS QDs and a relatively lower one for the reference FAPI films, as shown in Figure S5, in accordance with the slightly faster crystallization at low temperature experimentally observed. The observation of lower temperature for the formation of the black phase of FAPI perovskite film with the addition of embedded PbS QDs is in line with previous results for pure dimethysulfoxide (DMSO) solvent

system.[5] This means that also under air conditions, a strong interaction between the FA cation and the coordinating solvent (Lewis base), such as the NMP, due to the formation of a stable FAI.PbI_2.NMP adduct intermediate phase facilitates a more effective perovskite crystallization process than the conventional δ-FAPI intermediate pathways.[2] Unlike the DMSO, the quick separation of NMP solvent from the adduct,[2] along with the chemical and structural benefits of PbS QDs, helps the formation of α-FAPI under ambient conditions.

The XRD patterns of the FAPI (air) and perovskite films with FAPI-PbS QDs annealed at 165 °C, are compared in Figure S6a. The reference FAPI (air) and FAPI-PbS QDs mixed films (0.5, 1, 2.5, and 5 mg/mL) showed similar XRD diffraction patterns with a small shift for the (100) and (200) diffraction peaks (see Figure S6b,c for the Gaussian fit), confirming that the intrinsic perovskite crystalline phase is slightly distorted with the FAPI-PbS QDs inclusion.[5, 6] The FAPI-PbS QDs in perovskite matrix can generate a local strain that concomitantly promotes the chemical amalgamation between the perovskite α-phase with the (100) crystalline phase of PbS QDs.[5, 6, 10, 11] Table S1, shows the (100) and (110) XRD peak area comparison of the reference FAPI (air) and various amounts of FAPI-PbS QDs perovskite thin films. Higher intensity peak ratio is observed for the 0.5 and 1 mg/mL (air) films than the reference FAPI (air) film. This is symbolic of relatively more favored perovskite grain growth in the (100) plane induced by the PbS QDs.[6] Once the concentration overcomes 1 mg/ml the more nucleation seeds lead to smaller grains, more grain boundaries, in turn affecting the crystallinity.[5]

All the perovskite films showed a similar absorbance profile throughout the wavelength region and band edge region, Figure S6d. The bandgap of the reference FAPI (air) and various amount of FAPI-PbS QDs perovskite films are derived from Tauc plots (Figure S6f and Table S2), and each film shows a bandgap value of ~1.5 eV similar to the α-FAPI, confirming that the bandgap is not substantially affected despite the FAPI-PbS QDs inclusion. As shown in

Figure S6e, the presence of FAPI-PbS QDs did not appreciably change the emission wavelength, as expected from the similar bandgap observed.

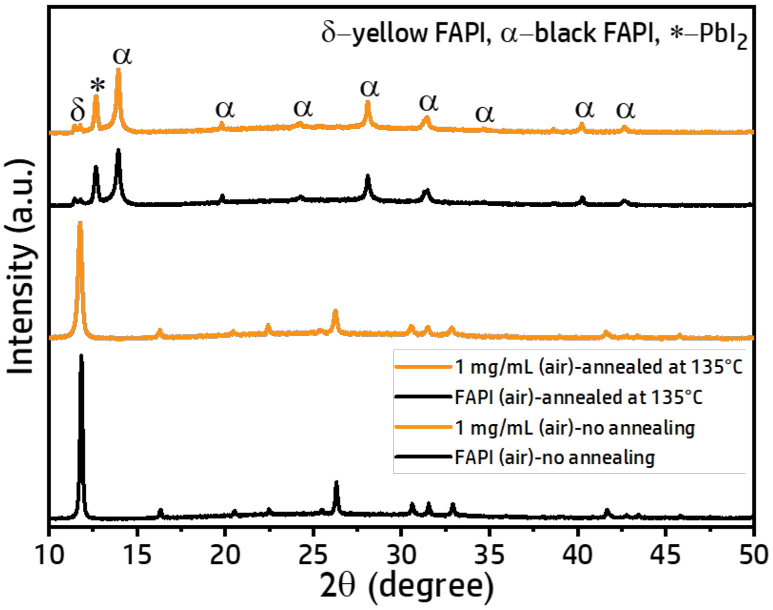

Figure S5. The comparative X-ray diffraction (XRD) patterns of the FAPI (air) and 1 mg/mL (air) perovskite thin film on the glass substrate, not annealed (just spun) and annealed at 135°C under an ambient atmosphere (at 25 °C, RH = 40-60%).

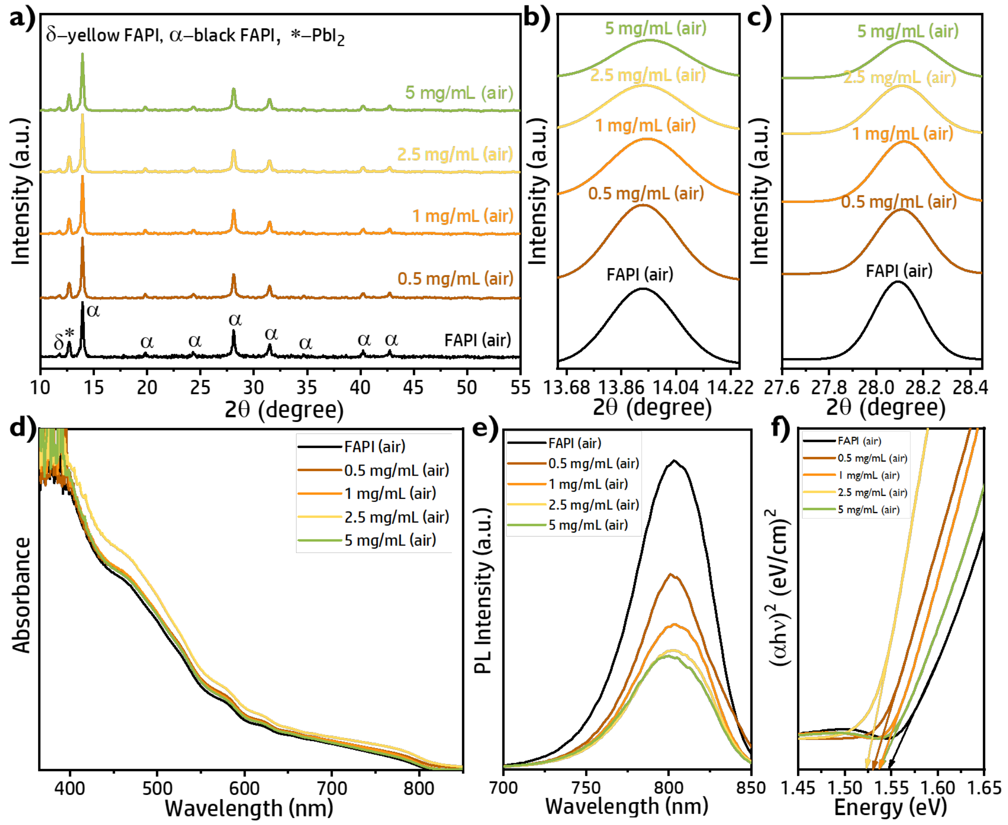

Figure S6. The structural and optical features of reference FAPI (air) with various amounts of FAPI-PbS QDs perovskite thin films. a) The X-ray diffraction (XRD) pattern. b, c) The Gaussian fit of the magnified (100) and (200) XRD peak; d) UV-vis absorption spectra, more details and discussion in Figure S24; e) Steady-state Photoluminescence (PL) spectra; f) The Tauc plots. The samples for XRD, UV-Vis absorption, and PL measurements are fabricated on glass substrates.

Table S1. The (100) and (110) XRD peak area comparison of the reference FAPI (air) and various amounts of FAPI-PbS QDs perovskite thin films.

**Sample**	(100) peak area	(110) peak area	(100)/(110) peak ratio
**FAPI (air)**	605.42	53.75	11.26
**0.5 mg/mL (air)**	596.38	51.20	11.65
**1 mg/mL (air)**	509.21	40.61	12.53
**2.5 mg/mL (air)**	428.03	58.69	7.29
**5 mg/mL (air)**	287.61	41.51	6.92

Table S2. The bandgap value of reference FAPI (air) and various amounts of FAPI-PbS QDs perovskite thin films derived from the Tauc Plots.

**Sample**	**The bandgap (eV)**
**FAPI (air)**	1.544
**0.5 mg/mL (air)**	1.530
**1 mg/mL (air)**	1.531
**2.5 mg/mL (air)**	1.523
**5 mg/mL (air)**	1.534

Table S3. The statistical parameters of the domain size distribution for reference FAPI (air) and various amounts of FAPI-PbS QDs perovskite thin films.

**Sample**	Mean size (nm)	Standard deviation	Minimum size (nm)	Maximum size (nm)
**FAPI (air)**	418	94	254	696
**0.5 mg/mL (air)**	567	126	300	824
**1 mg/mL (air)**	646	118	426	850
**2.5 mg/mL (air)**	456	53	346	539

**5 mg/mL (air)**	363	87	361	520

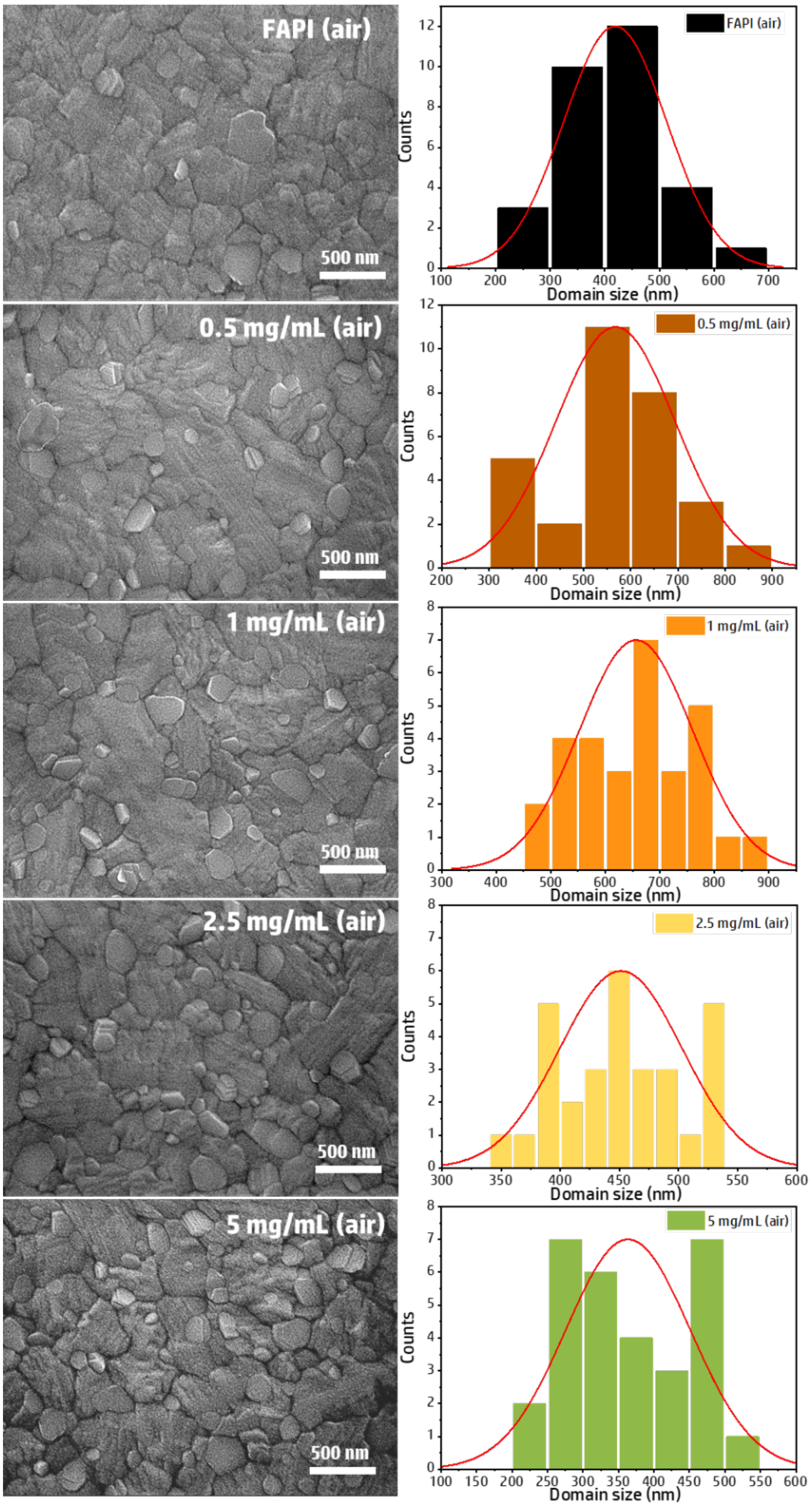

Figure S7. Scanning Electron Microscopy (SEM) top-view images of reference FAPI (air) and various amounts of FAPI-PbS QDs perovskite thin films with the histogram of perovskite domain sizes visible

by SEM, the scale bar is 500 nm. The samples for SEM measurements are fabricated on conductive ITO substrates. The statistical parameters are given in Table S3.

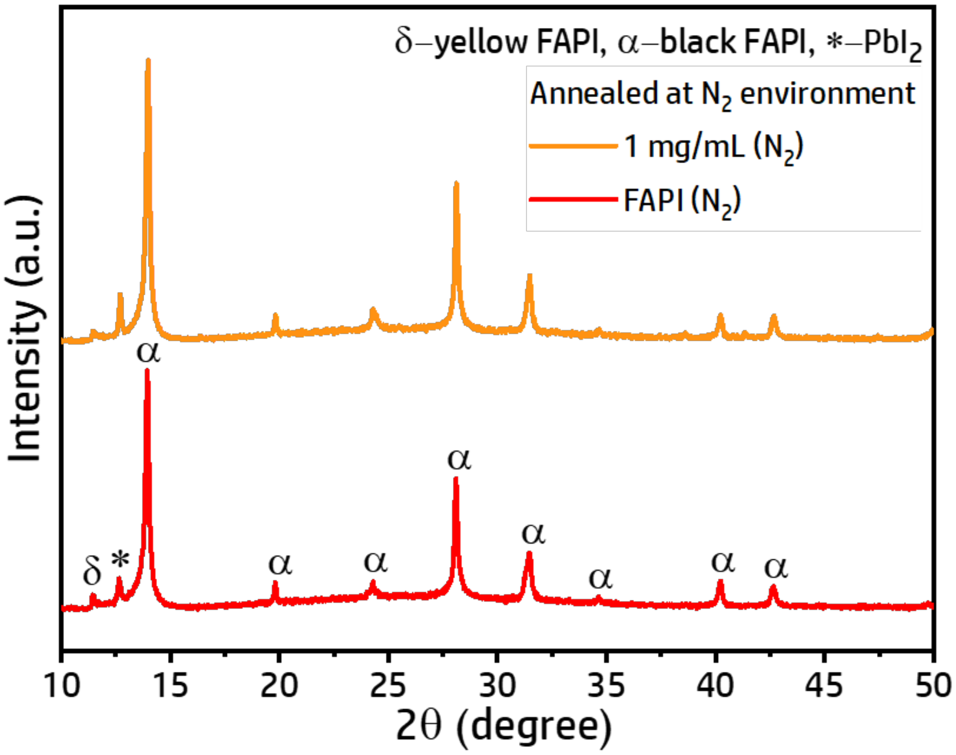

Figure S8. The X-ray diffraction (XRD) patterns of the FAPI (N_2) and 1 mg/mL (N_2) perovskite thin film prepared and annealed under N_2 environment.

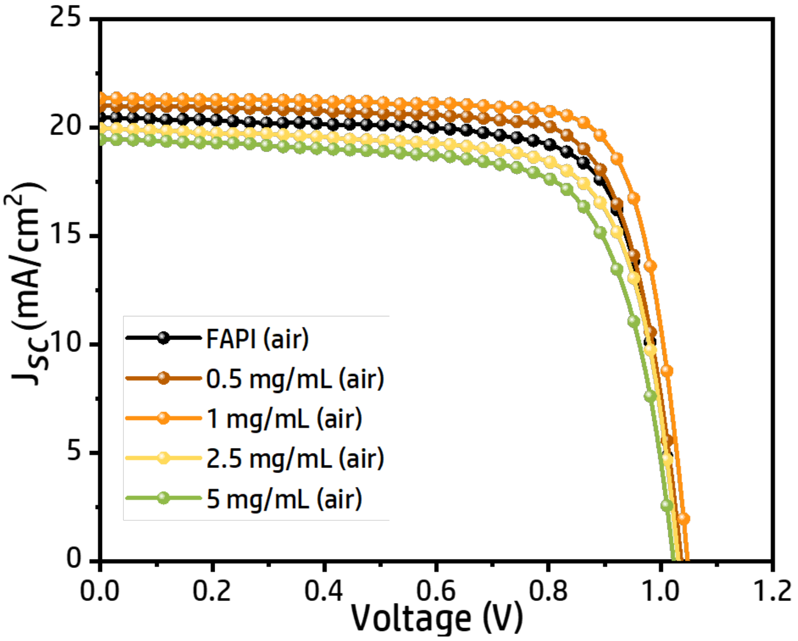

Figure S9. The current-voltage (J-V) curves of reference FAPI (air) and various amounts of FAPI-PbS QDs device are recorded at reverse bias scan direction (from 1.2 V to -0.02 V) with a scan rate of 10 mV/s. The solar cells were measured under a simulated air mass 1.5 global (AM 1.5G) of 1 Sun illumination (irradiation intensity of 1000 Wm^{-2}).

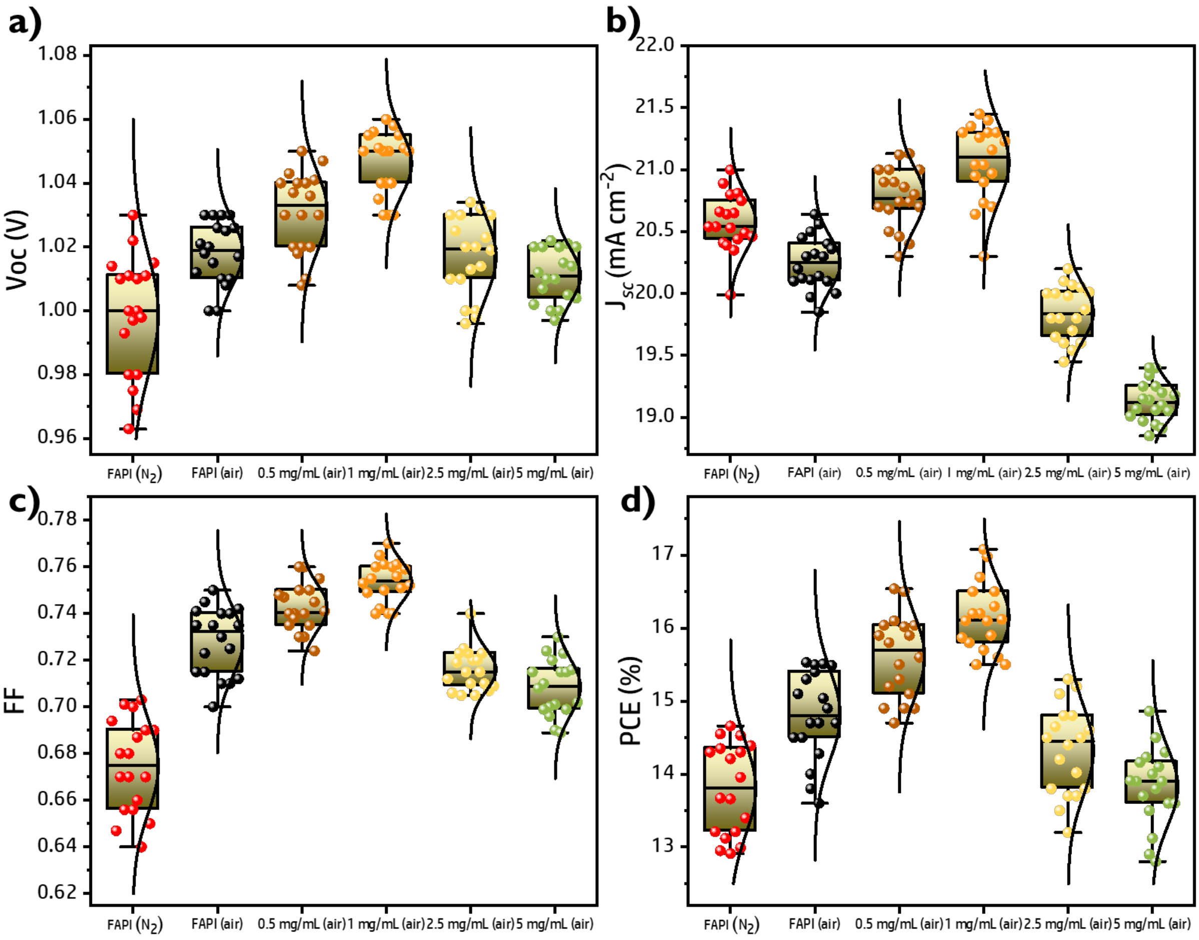

Figure S10. The statistical distribution of photovoltaic features of 18 devices for reference FAPI (N_2 and air) and various amounts of FAPI-PbS QDs, the device parameters like; a) V_{oc}, b) J_{sc}, c) FF, and d) PCE. The performance of the 0.5 mg/mL FAPI-PbS QDs device is moderately better than that obtained for the reference FAPI (air and N_2); showing a champion PCE of 16.54 %. However, as the mixing of FAPI-PbS QDs increased beyond 1 mg/mL (air), the device performances are slowly decreased for 2.5 and 5 mg/mL (air), mainly the drops in V_{oc}, J_{sc}, and FF, and the devices achieved a champion PCE of 15.31 % and 14.86 % respectively. The presence of a higher amount than 1 mg/mL of FAPI-PbS QDs in the FAPI matrix resulted in deleterious effects on charge transport and radiative recombination, in line with the PL, lower crystal orientation, and smaller grain size (Figure S7).

Table S4. The photovoltaic performance characteristics of solar cells of the reference FAPI (N_2 and air) and different amounts of FAPI-PbS QDs. The average photovoltaic characteristics of solar cells including the standard deviations of the open-circuit voltage (V_{oc}), short-circuit current density (J_{sc}), and fill factor (FF). The champion cell efficiency of reference FAPI (N_2 and air) and FAPI-PbS QDs are given in the brackets. The average statistical distribution is based on 18 solar cells on different substrates for each amount of FAPI-PbS QDs.

Sample	V_{oc} (V)	J_{sc} (mA cm^{-2})	FF	PCE (%)
**FAPI (N_2)**	1.00±0.03 **(1.03)**	20.63±0.40 **(20.95)**	0.65±0.02 **(0.67)**	14.10±0.34 **(14.36)**
**FAPI (air)**	1.02±0.02 **(1.03)**	20.32±0.50 **(20.56)**	0.73±0.02 **(0.75)**	14.95±0.51 **(15.53)**
**0.5 mg/mL (air)**	1.03±0.02 **(1.05)**	20.95±0.40 **(21.13)**	0.74±0.02 **(0.76)**	16.08±0.40 **(16.54)**
**1 mg/mL (air)**	1.05±0.02 **(1.06)**	21.21±0.40 **(21.43)**	0.75±0.02 **(0.77)**	16.56±0.30 **(17.08)**
**2.5 mg/mL (air)**	1.02±0.01 **(1.03)**	19.75±0.50 **(20.07)**	0.72±0.03 **(0.74)**	14.81±0.52 **(15.31)**
**5 mg/mL (air)**	1.01±0.01 **(1.02)**	19.05±0.30 **(19.24)**	0.71±0.02 **(0.73)**	14.12±0.56 **(14.86)**

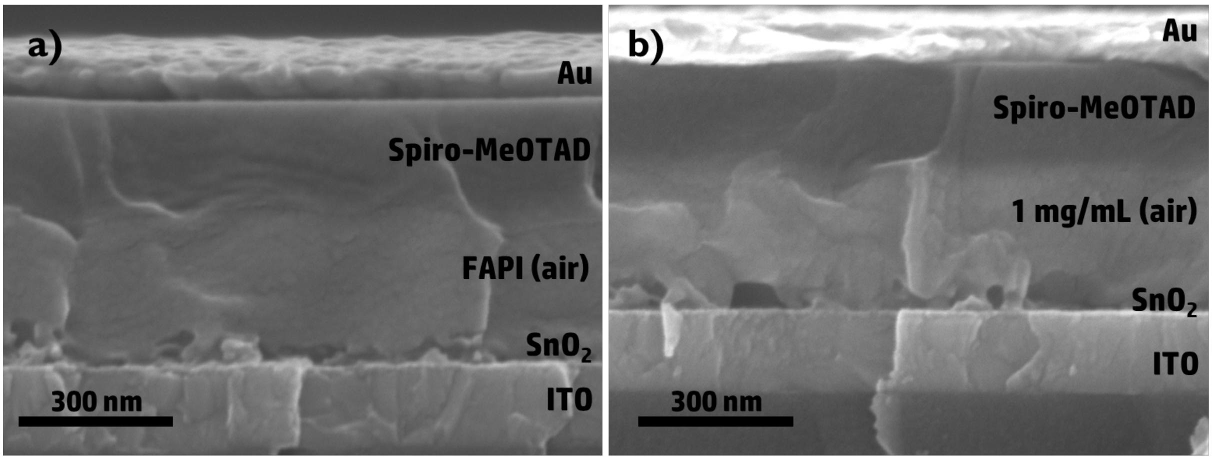

Figure S11. The cross-sectional scanning electron microscopy images of perovskite solar cell prepared with a) reference FAPI (air); b) 1 mg/mL (air). The device layer consists of Indium tin oxide (ITO)/SnO_2/Perovskite (FAPI (air) or 1 mg/mL (air)/2,2',7,7'-tetrakis-(N,N-di-4-methoxyphenylamino)-9,9'-spirobifluorene (Spiro-MeOTAD)/Au.

Table S5. The photovoltaic parameters of perovskite solar cells based on reference FAPI (N_2 and air) and 1 mg/mL (air) under 1 sun illumination (AM 1.5 G, 1000 Wm^{-2}). All the devices are recorded at forward and reverse bias scan direction (-0.02 V to 1.2 V and vice versa) with a scan rate of 10 mV/s. The champion cell efficiency is given in the brackets and the average statistical distribution is based on 18 solar cells.

Sample	Scan direction	V_{oc} (V)	J_{sc} (mA cm^{-2})	FF	PCE (%)	Best PCE (%)
**FAPI (N_2)**	reverse scan	1.00±0.03 **(1.03)**	20.63±0.40 **(20.95)**	0.65±0.02 **(0.67)**	14.10±0.34 **(14.36)**	14.36
	forward scan	0.98±0.02 **(0.99)**	20.50±0.30 **(20.80)**	0.63±0.02 **(0.62)**	12.65±0.20 **(12.55)**	12.55
**FAPI (air)**	reverse scan	1.02±0.02 **(1.03)**	20.32±0.50 **(20.56)**	0.73±0.02 **(0.75)**	14.95±0.51 **(15.53)**	15.53
	forward scan	1.00±0.02 **(1.00)**	20.15±0.30 **(20.09)**	0.72±0.01 **(0.71)**	14.35±0.40 **(14.25)**	14.25
**1 mg/mL (air)**	reverse scan	1.05±0.02 **(1.06)**	21.21±0.40 **(21.43)**	0.75±0.02 **(0.77)**	16.56±0.30 **(17.08)**	17.08
	forward scan	1.05±0.01 **(1.05)**	21.05±0.20 **(21.22)**	0.75±0.01 **(0.76)**	16.45±0.25 **(16.92)**	16.92

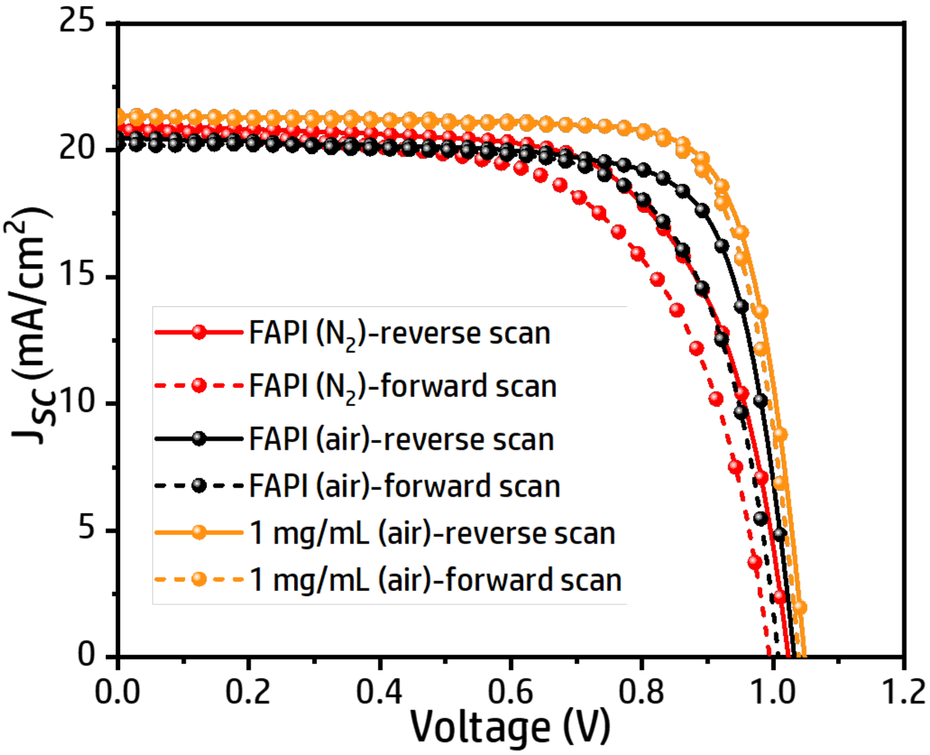

Figure S12. The current-voltage (J-V) curves of reference FAPI (N_2 and air) and 1 mg/mL (air) device recorded at forward and reverse bias scan direction (-0.02 V to 1.2 V and vice versa) with a scan rate of 10 mV/s; the solar cells were measured under a simulated air mass 1.5 global (AM 1.5G) of 1 Sun

illumination (irradiation intensity of 1000 Wm^{-2}). The lower amount of hysteresis is observed in the case of the FAPI (air) and 1 mg/mL (air) device (with the hysteresis index of 0.08 and 0.008) compared to the FAPI (N_2) device (with the hysteresis index of 0.12).

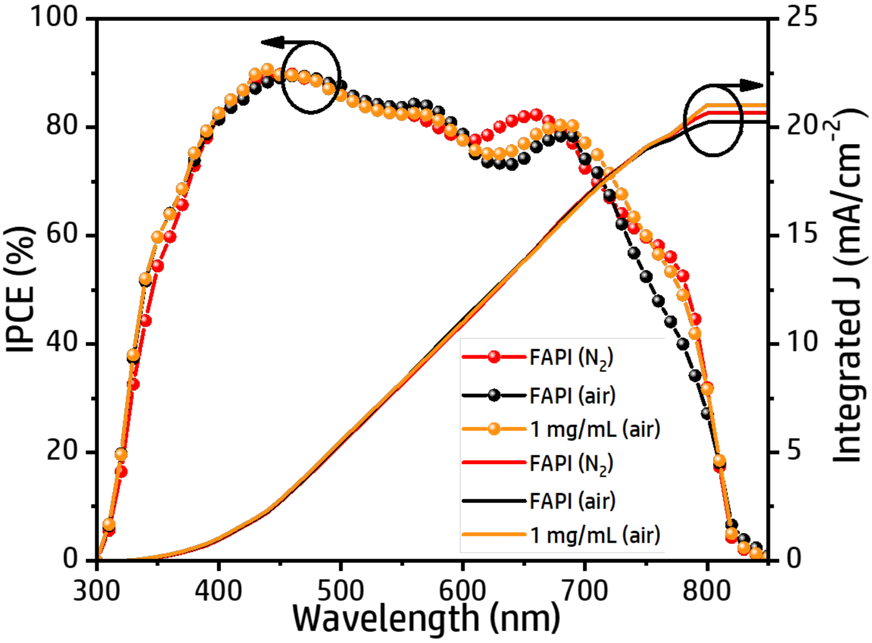

Figure S13. The IPCE spectra and integrated current density of reference FAPI (N_2 and air) and 1 mg/mL (air) device. The lower IPCE at longer wavelengths (750-800 nm) for FAPI (air) film is due to the lower absorption in this region, see Figure 1a.

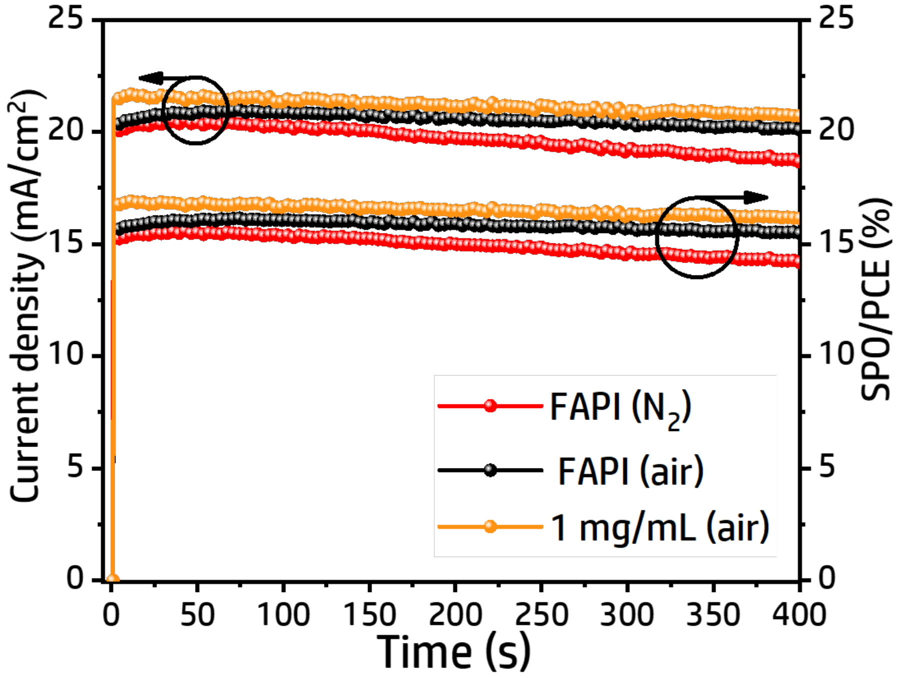

Figure S14. The stabilized photocurrent density at maximum power point vs time for FAPI (air, N_2) and 1 mg/mL (air) devices. The photocurrent at maximum power point for the reference FAPI (air and N_2) and 1 mg/mL (air) devices at maximum power point photovoltage for a short-term at ambient air (relative humidity of ~60%), which shows that the FAPI-PbS QDs has an important role in the device performance stability.[5, 6] Moreover, it is observed that

the device 1 mg/mL (air) reaches the maximum power point (with a stabilized PCE of 16.87%) in few seconds and that for reference FAPI (air and N_2) device takes more time.

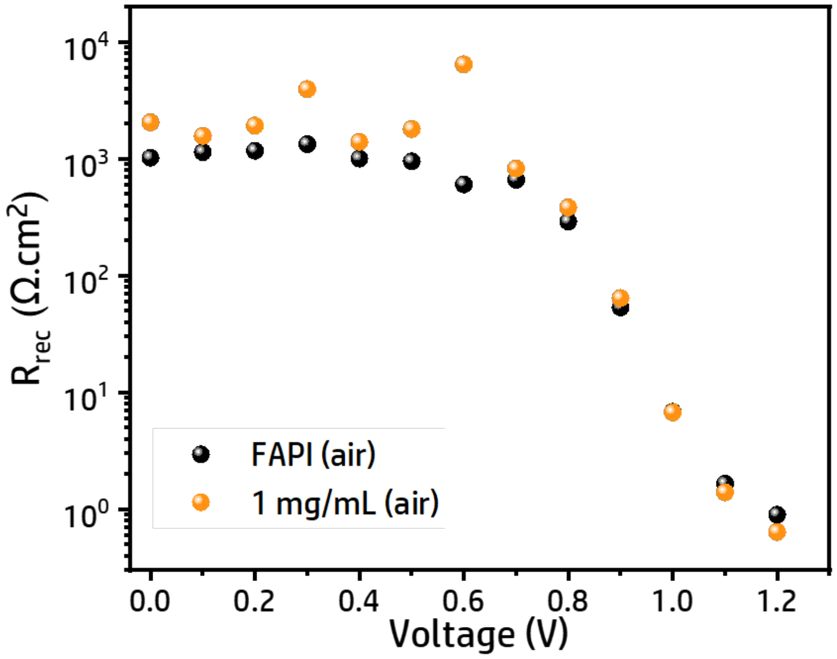

Figure S15. The plot of recombination resistance (R_{rec}) versus applied voltage under 1 sun illumination was obtained from the fitting of EIS using the equivalent circuit previously reported.[4] Recombination resistance is similar in both devices, presenting slightly higher values for 1 mg/mL (air).

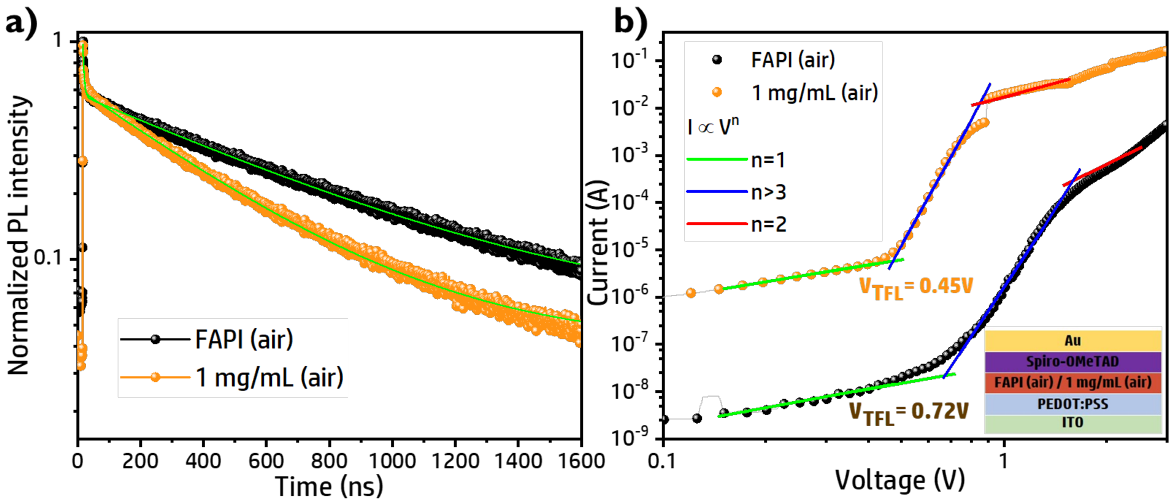

Figure S16. A) The time-resolved photoluminescence spectra of reference FAPI (air) and 1 mg/mL (air) films prepared on glass substrates; b) The dark current-voltage (I–V) characteristics of the electron-only device (ITO/SnO_2/FAPI (air) perovskite (and 1 mg/mL (air))/PCBM/Au) for the SCLC measurements. The dark I−V has three characteristic regions: (i) an Ohmic region at low voltage range (green line), (ii) the trap-filled limited region (V_{TFL}) at intermediate voltage range where the current abruptly increases (blue line), and (iii) a trap-free region at high voltage range SCLC region (red line). The trap density (n_{trap}) is calculated by the equation, $n_{trap} = (2\varepsilon_r\varepsilon_0 V_{TFL})/(eL^2)$, where n_{trap} is the trap density, ε_r is the dielectric constants of perovskite, ε_0 is the permittivity of vacuum, L is the thickness of the perovskite film, e is the elementary charge and V_{TFL} is the

trap-filled limit voltage acquired by fitting the dark I−V data. The V_{TFL} values are found to be 0.72 V and 0.45 V for the reference FAPI (air) and 1 mg/mL (air) films, respectively. The reference FAPI (air) device shows a higher trap density of 3.8×10^{16} cm^{-3}, than the trap density of 2.1×10^{16} cm^{-3} of the 1 mg/mL (air).

Table S6. The arbitrary data used to fit the time-resolved photoluminescence curves for the reference FAPI (air) and 1 mg/mL FAPI-PbS QDs perovskite thin film. The PL decay curves are fitted with a bi-exponential equation as given below. Where A_1 and A_2 are the relative amplitudes and τ_1 and τ_2 are the decay times. $D(t) = A_1 \exp(-t/\tau_1) + A_2 \exp(-t/\tau_2) + C$

Sample	A_1	τ_1 (ns)	A_2	τ_2 (ns)	$<\tau_{avg}>$* (ns)
FAPI (air)	0.055	9.82	0.945	679.15	678
1 mg/mL (air)	0.077	7.97	0.923	531.43	531

$<\tau_{avg}>$* is the average lifetime; $<\tau_{avg}> = (A_1\tau_1^2 + A_2\tau_2^2)/(A_1\tau_1 + A_2\tau_2)$,[12] whereas $(A_1+A_2) = 1$.

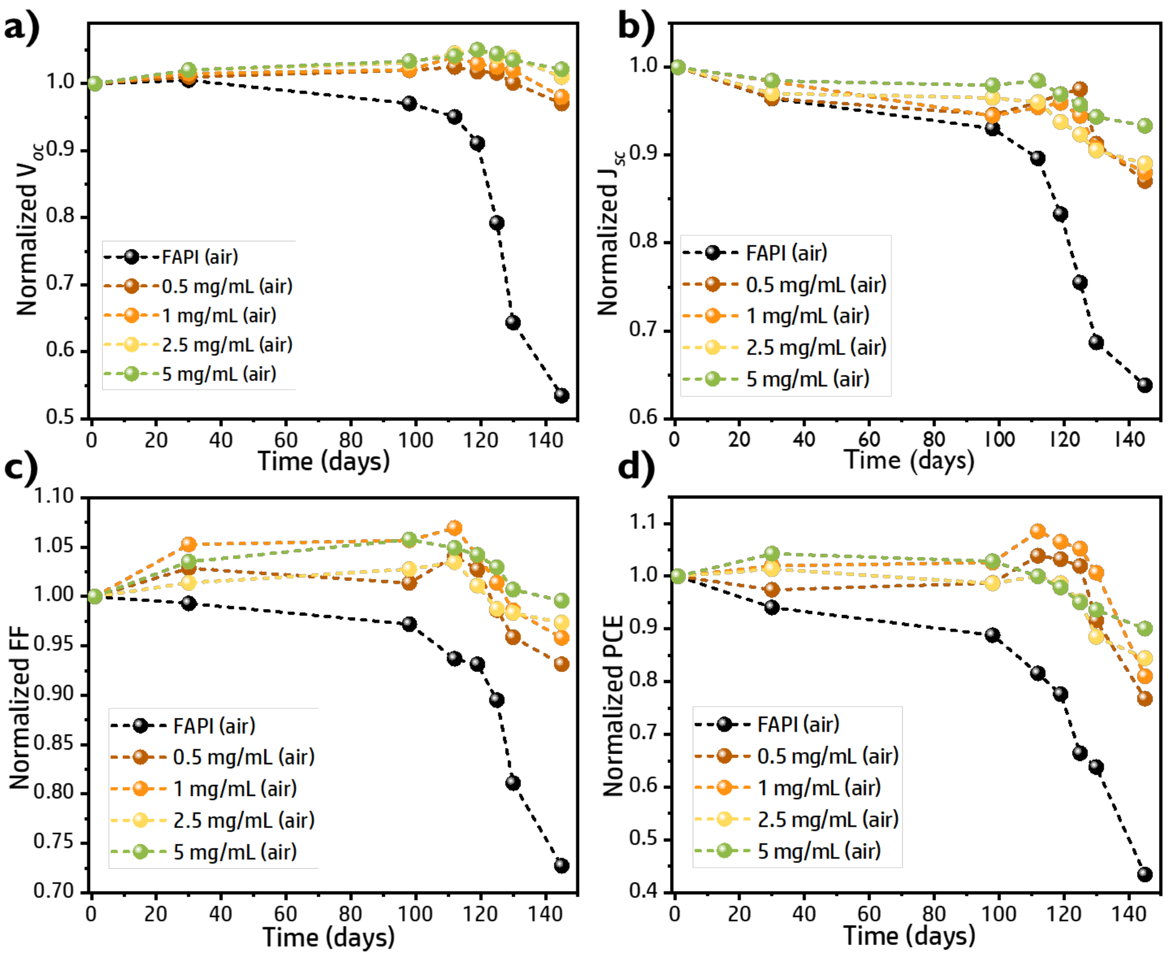

Figure S17. The long-term stability (145 days) comparison of the unencapsulated reference FAPI (air) and various amount of FAPI-PbS QDs devices (the data obtained from the average of 5 different devices) the normalized photovoltaic parameters like a) open-circuit voltage (V_{OC}); b) Short-circuit current density (J_{SC}); c) fill factor (FF), and d) power conversion efficiency (PCE), with a relative humidity of 23% and 25°C, at dark condition.

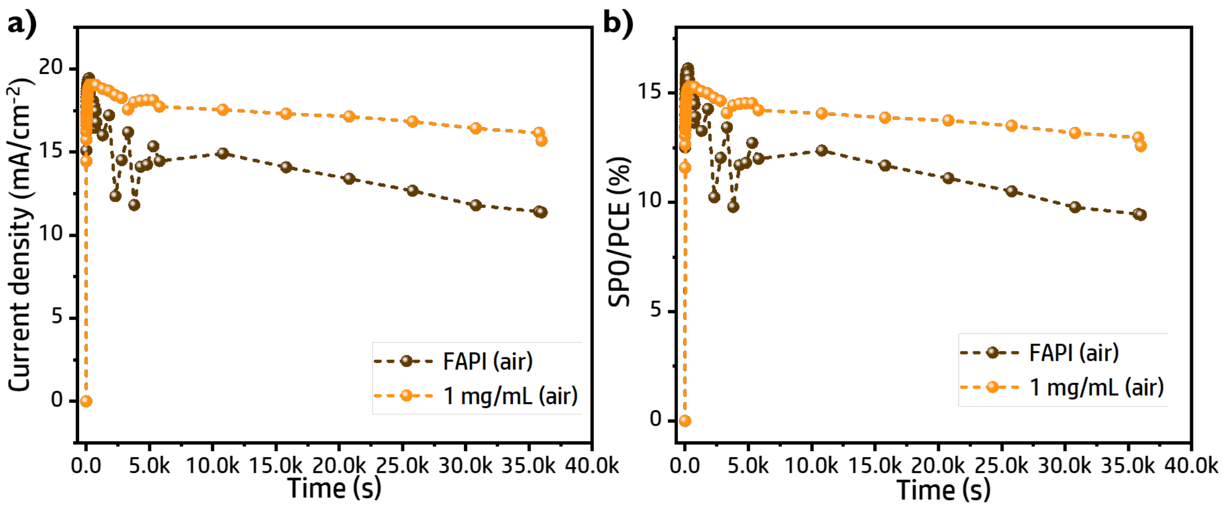

Figure S18. The photostability test of unencapsulated perovskite devices at 25°C and RH 60% (10 hours each); a) the stabilized photocurrent density and b) PCE at maximum power point vs time for the reference FAPI (air) and 1 mg/mL (air) devices. Measurements were carried out at 25ºC in the air atmosphere with RH 60%. In detail, the unencapsulated FAPI (air) based device efficiency drops to 42% from its initial value, while the device with 1 mg/mL (air) is found to be stable and a relatively lower amount of degradation is observed, retained >83% of the device efficiency. It is also observed that the device with FAPI-PbS QDs (air) reaches the maximum power point in few seconds and that for reference FAPI (air) device takes more time; this also signifies the better quality of perovskite layer and mitigated hysteresis due to the FAPI-PbS QDs inclusion.[13]

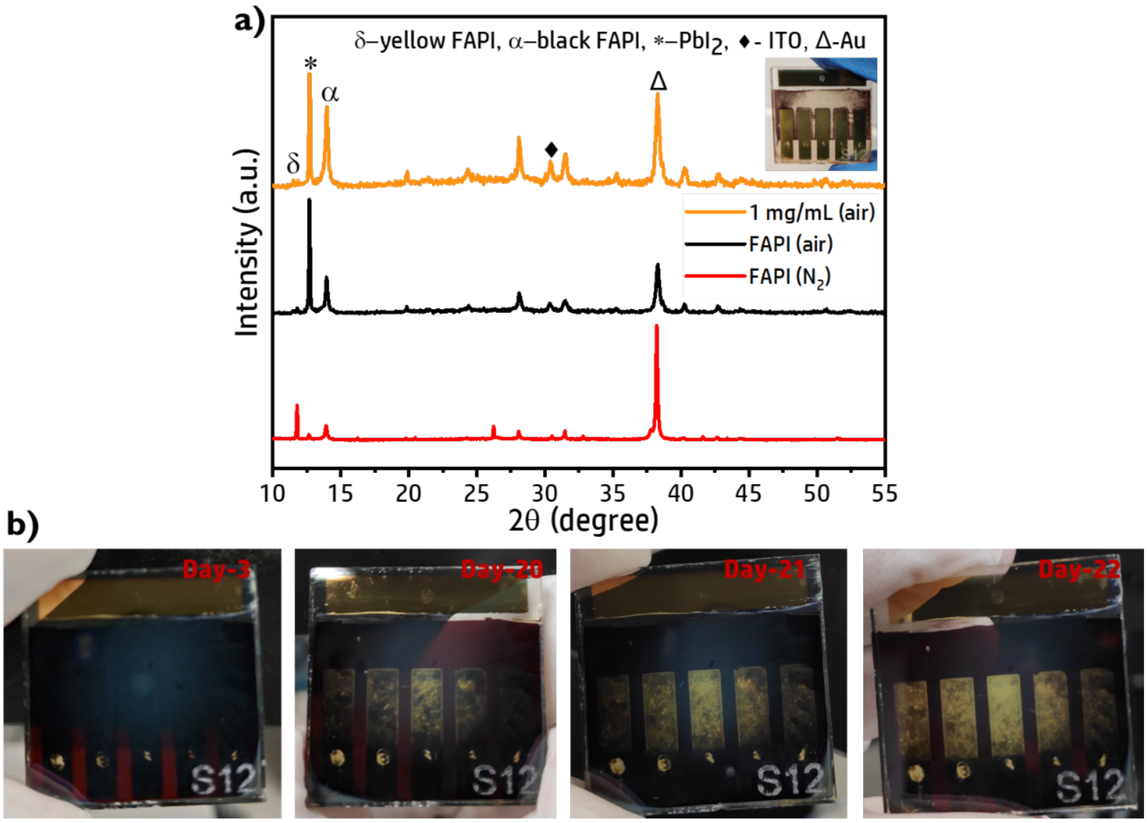

Figure S19. a) The XRD patterns of the aged perovskite solar cells with gold contact (devices were stored at ambient conditions of 25 °C and RH of 23%, at the dark condition). Note that the diffraction peak denoted as (♦) is from the ITO substrate and (Δ) is from the gold contact layer. The inset shows the figure of FAPI (N_2) device after one month; b) Photographic images of FAPI (N_2) devices with time (the devices were stored in ambient air without encapsulation, 25 °C, RH of 23%, at the dark condition).

Stability measurements with PbS QDs

In order to investigate the influence of FAPI-PbS QDs towards the tolerance of moisture, we evaluated the stability of perovskite thin films of reference FAPI (air) and FAPI-PbS QDs in the FAPI matrix (on glass substrate without any encapsulation) by monitoring the optical and structural features under ambient conditions with RH of 40±10 % and 25 °C, at the dark. Initially, we observed the changes of XRD patterns of the fresh and aged films of reference FAPI (air) and with the inclusion of FAPI-PbS QDs films. As shown in Figure S20, the degradation of reference FAPI (air) film started with the formation of the PbI_2 phase (12.70°) and converted to the δ-FAPI phase (11.80°) in a week.[2, 3]

Subsequently, the FAPI (air) film completely transformed into a yellow phase in two weeks with the signature of δ-FAPI peaks of 11.80° (010) and 26.30° (021). However, the degradation pathway of the perovskite samples with FAPI-PbS QDs is different than the reference FAPI (air) films. As shown in Figure S20, all the films (0.5, 1, 2.5, and 5 mg/mL (air)) started to degrade in PbI_2 (12.70°) and a negligible amount of δ-FAPI (11.80°). As shown in Figure S20-S22, the comparative analysis between the SEM images, thin-film photographs, and the XRD patterns clearly show the formation of small PbI_2 crystallites in all the perovskite films and for the aged perovskite film the appearance of the δ-FAPI phase (yellow phase) for the reference FAPI (air) films. While, only the reduction of the α-phase with the formation of PbI_2 as degradation products observed for the films of FAPI-PbS QDs, without the complete conversion to the delta phase, Figure S20, and S21. This corroborates with the SEM measurements of the representative films after the aging process, see Figure S22.

The stability of perovskite films is furthermore verified with the absorbance spectroscopy. As shown in Figure S25 the UV-Vis absorbance spectra of reference FAPI (air) film is remarkably reduced after aging for 10 days with the appearance of the photoinactive yellow perovskite material in 14 days. On the contrary, the absorption spectra of FAPI-PbS QDs films are showed only a slight variation in absorbance after aging for 10 days, shown in Figure S25. Moreover, the samples with a higher amount of FAPI-PbS QDs (eg, 2.5 and 5 mg/mL (air)) films showed better absorbance and stability than the films with a lower amount of FAPI-PbS QDs counterparts (as shown in Figure S25). More evidently, the FAPI-PbS QDs induces a strain which constitutes the compression of the unit cell (see Figure S6b, c) and consecutive α phase stabilization through chemical interactions.[5, 6, 11]

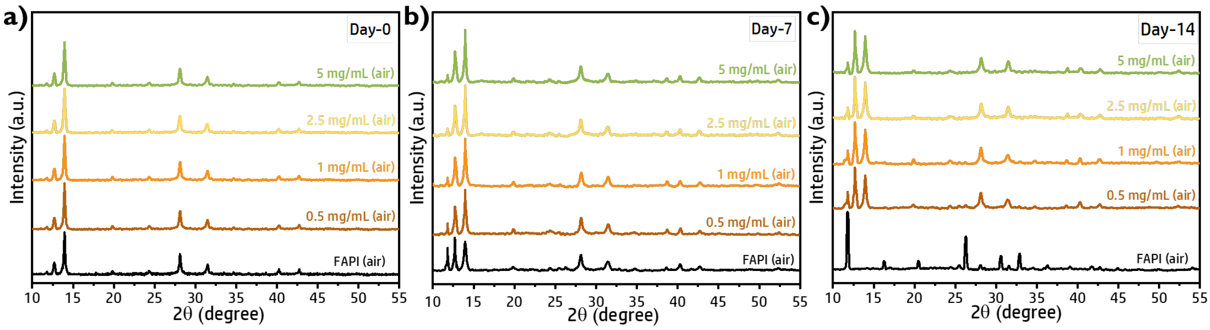

Figure S20. The structural features (XRD patterns) of 14 days aged perovskite thin films of reference FAPI (air) and various amounts of FAPI-PbS QDs fabricated on glass substrates (the samples are stored under an ambient atmosphere with a relative humidity of 40±10 % and 25°C, at dark).

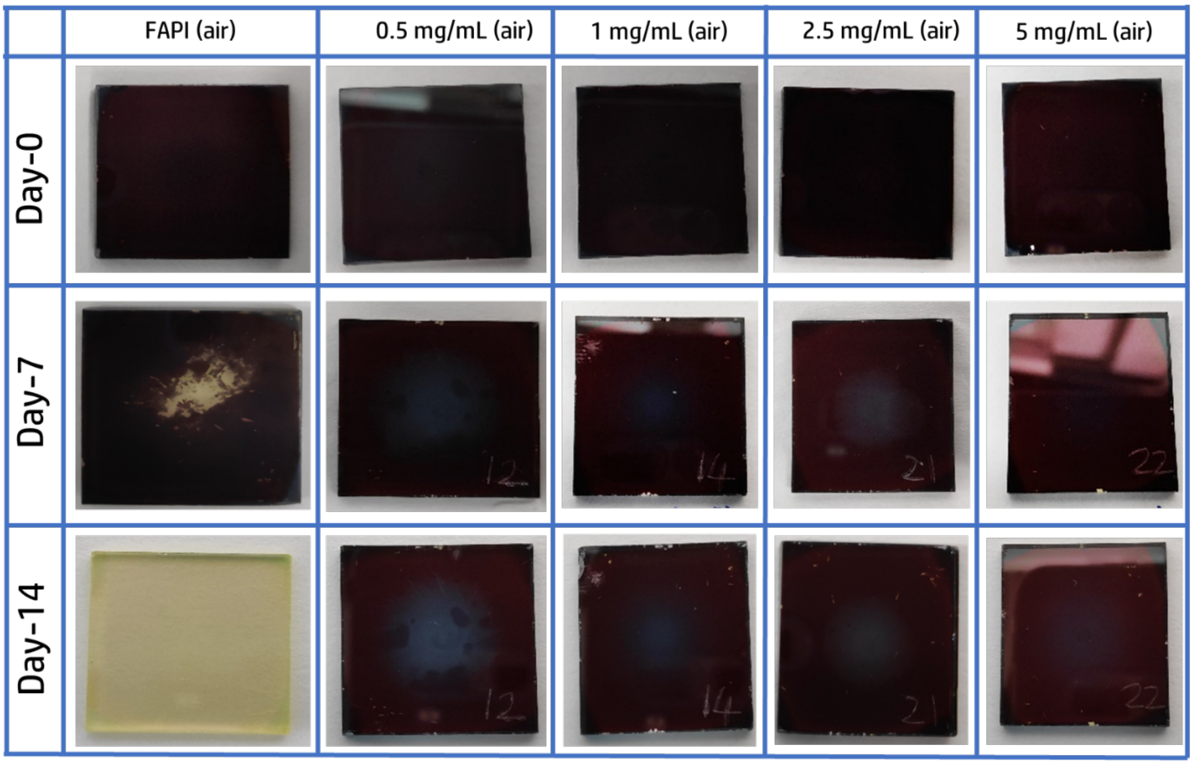

Figure S21. The photographic images of 14 days aged perovskite thin films of reference FAPI (air) and various amounts of FAPI-PbS QDs fabricated on glass substrates (the samples are stored under an ambient atmosphere with a relative humidity of 40±10 % and 25°C, at dark).

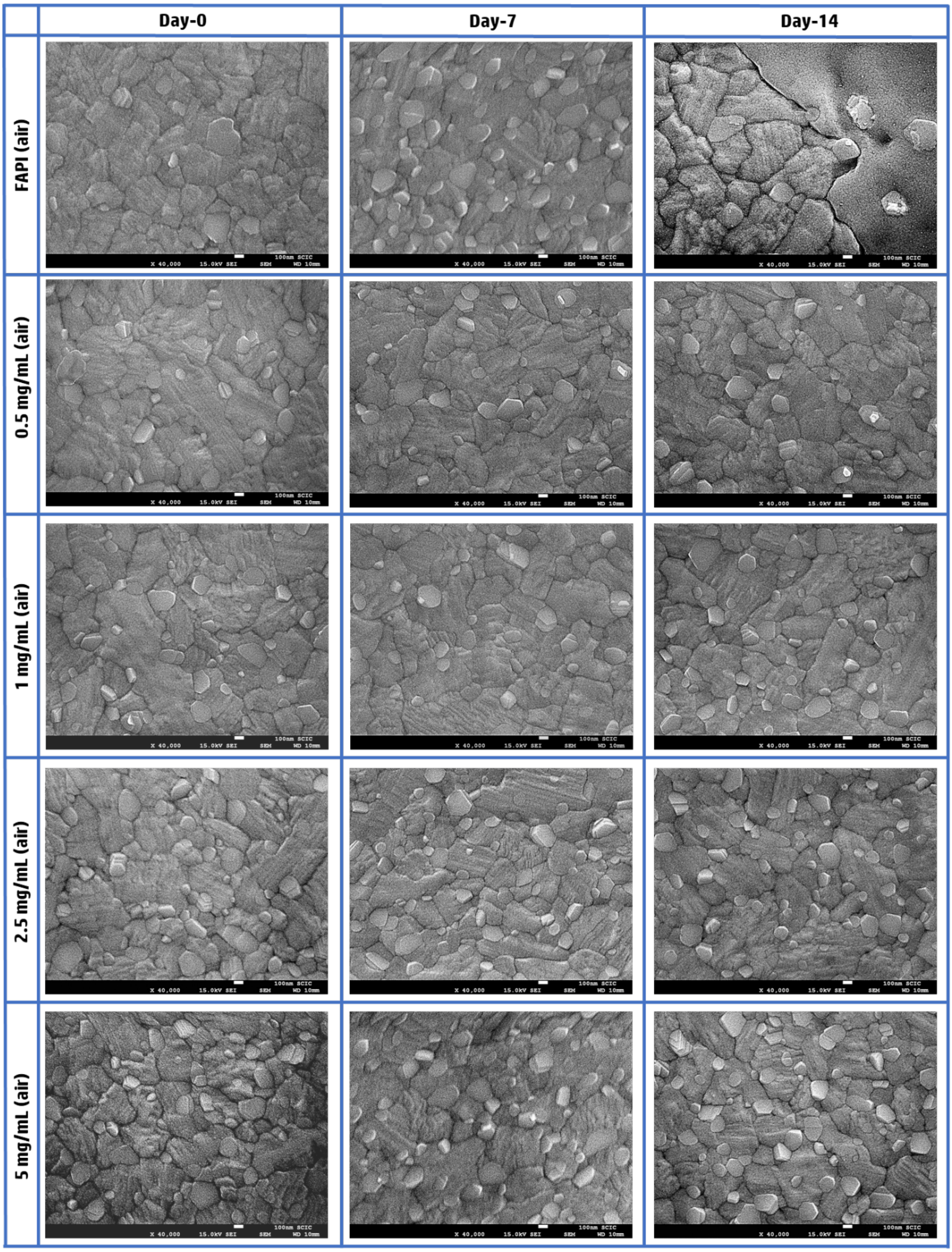

Figure S22. The SEM micrographs of 14 days aged perovskite thin films of reference FAPI (air) and various amounts of FAPI-PbS QDs fabricated on ITO substrates (the scale bar is 100 nm, the samples are stored under an ambient atmosphere with a relative humidity of 40±10 % and 25°C, at dark).

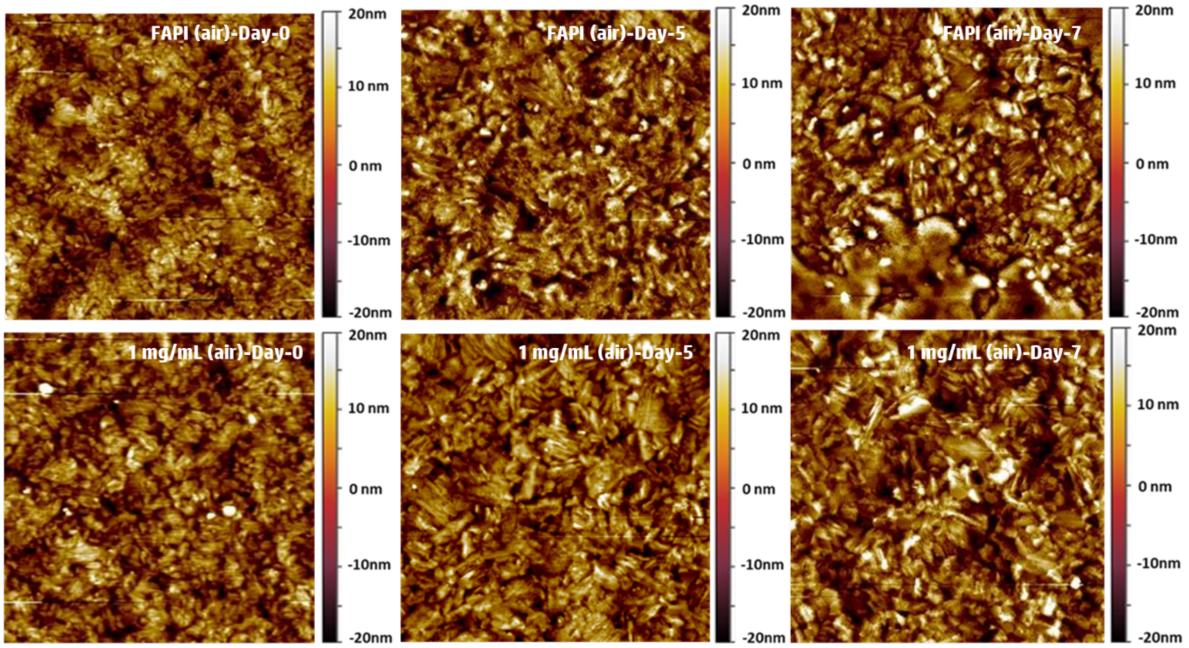

Figure S23. Atomic Force Microscopy (AFM) topography of aged perovskite thin films of reference FAPI (air) and 1 mg/mL (air) fabricated on glass/ITO substrates (the samples are stored under an ambient atmosphere with an RH of 40±10% and 25°C, at dark, without encapsulation, the scanned area is 10 x 10 μm^2).

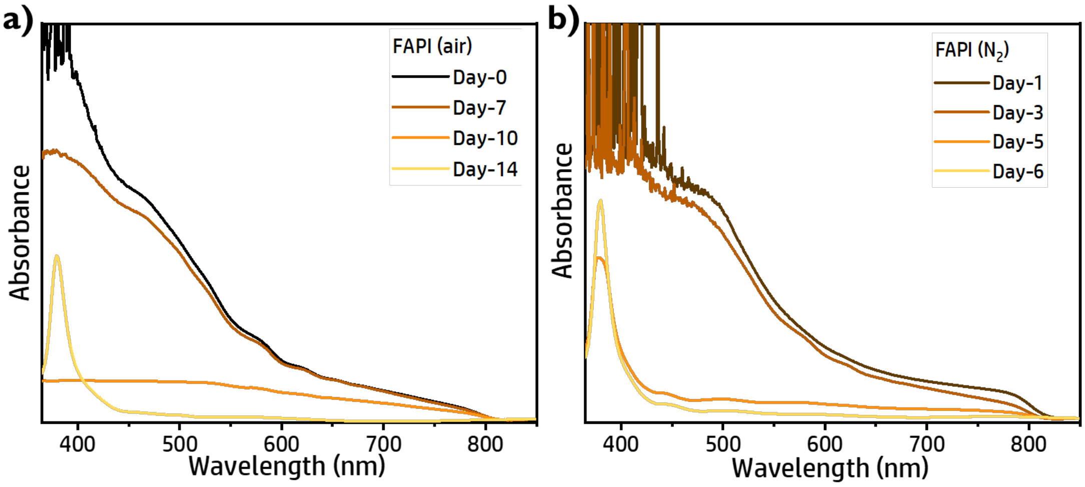

Figure S24. The UV-Vis absorbance spectra of aged perovskite thin films of FAPI (air) and FAPI (N_2) fabricated on glass substrates (the samples are stored under an ambient atmosphere with a relative humidity of 40±10 % and 25°C, at dark). Note that two shoulders at 570 nm and 620 nm are observed for FAPI (air) and significantly lower absorbance is measured for FAPI (air) than for FAPI (N_2) at the longer wavelength range (750-800 nm). In as deposited (Day-0) the FAPI (air) films shows a higher content of δ-FAPI and PbI_2 phases, responsible of the shoulders, than FAPI (N_2), see Figure 1b. The presence of these phases also is the responsible of the relatively lower of absorbance (in the longer wavelength range of 750-800 nm) observed for the FAPI (air) film when compared to the FAPI (N_2) film in Day-0. However, note that these effect, shoulders and lower absorption at 750-800 nm range is also observed for FAPI (N_2) Day-3 when the presence of δ-FAPI and PbI_2 phases has increased.

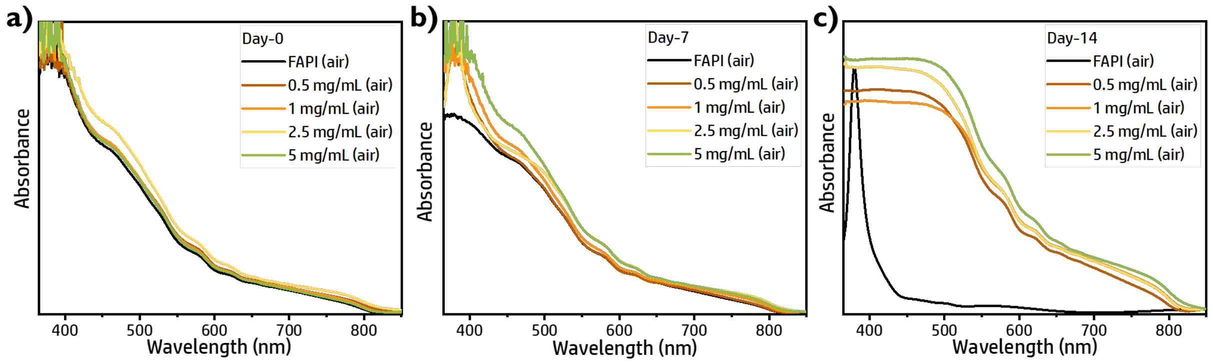

Figure S25. The UV-Vis absorbance spectra of 14 days aged perovskite thin films of reference FAPI (air) and various amounts of FAPI-PbS QDs fabricated on glass substrates (the samples are stored under an ambient atmosphere with a relative humidity of 40±10 % and 25°C, at dark).

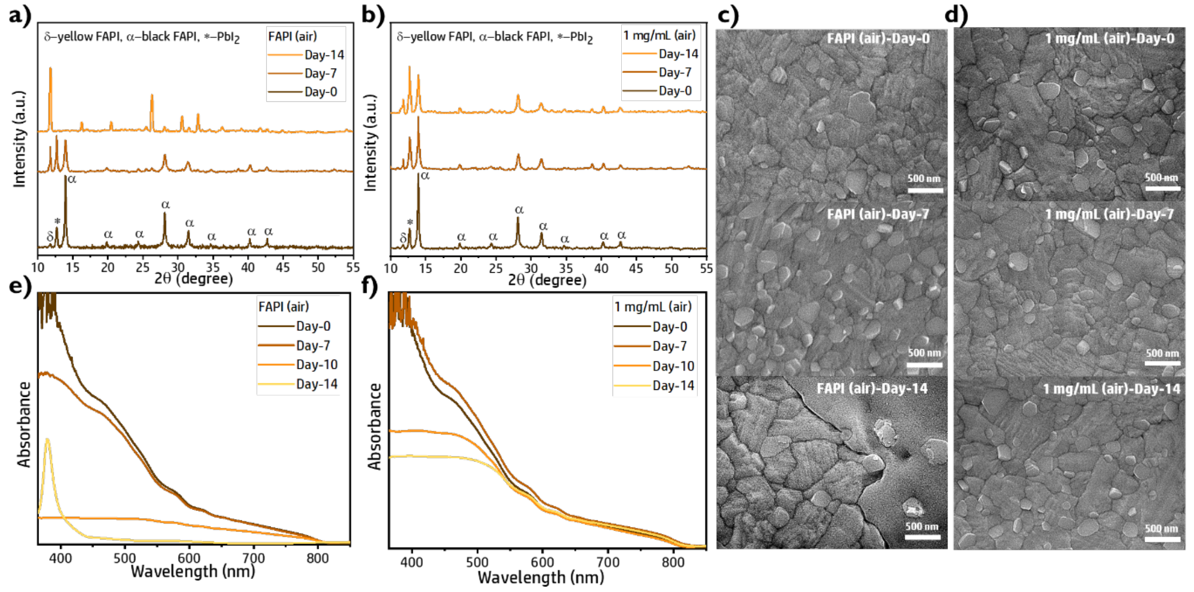

Figure S26. The structural, morphological, and optical features of 14 days aged perovskite thin films of reference FAPI (air) and 1 mg/mL (air) fabricated on glass/ITO (for SEM) substrates (the samples are stored under an ambient atmosphere with an RH of 40±10% and 25°C, at dark, without encapsulation), a and b) The X-ray diffraction pattern; c and d) SEM micrographs, the scale bar is 500 nm; e and f) UV-vis absorption spectra.

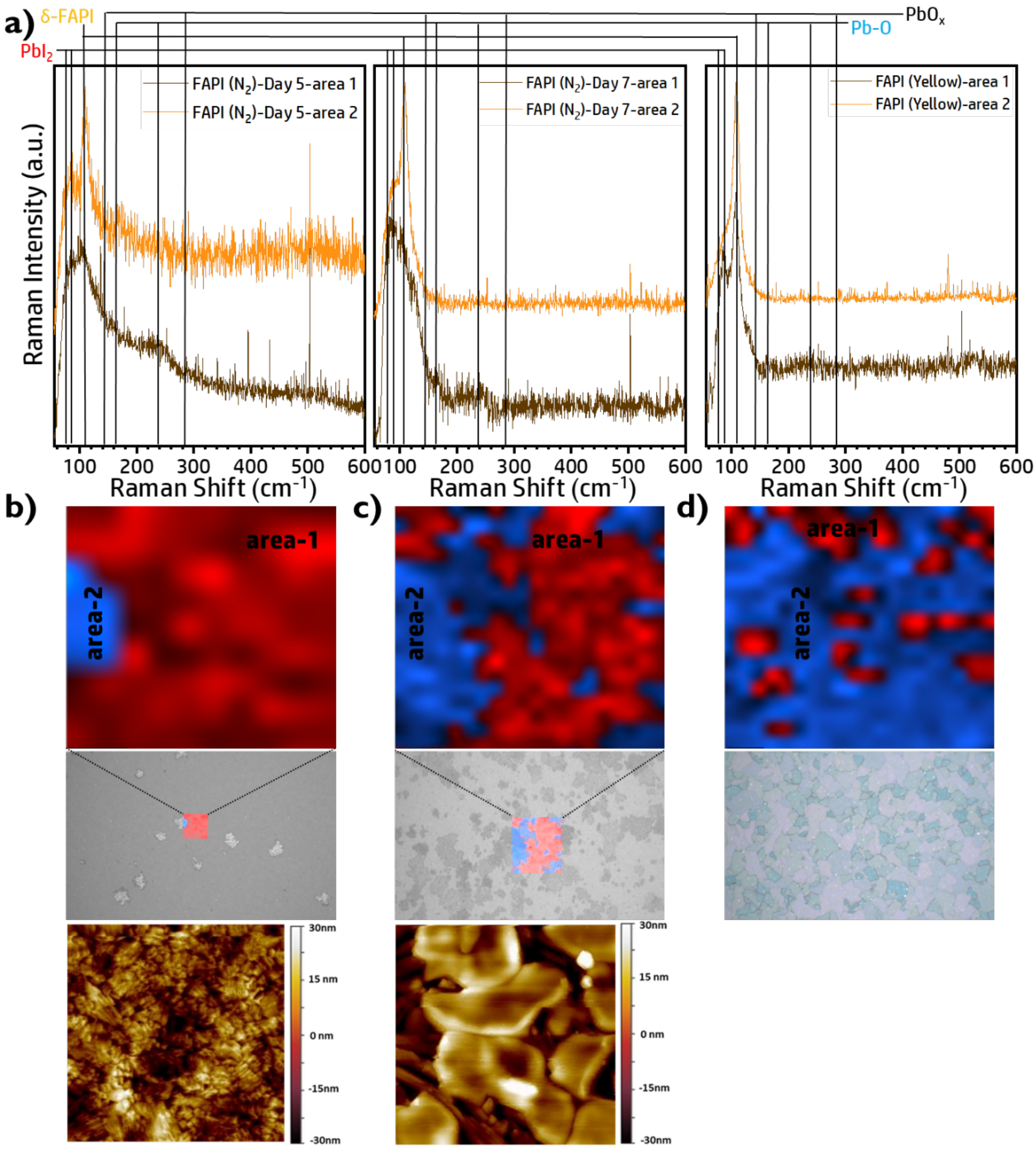

Figure S27. a) Raman Spectra of aged FAPI (N_2) films (at Day 5 and 7) and completely degraded film (δ-FAPI, indicated as yellow)) measured with a 0.1 mW power intensity; the presence of multiple spectral mode/components in the film is marked as area 1 and area 2. b, c, and d) are the corresponding optical and AFM images of the FAPI (N_2) films at Day-5, Day-7, and yellow film (δ-FAPI).

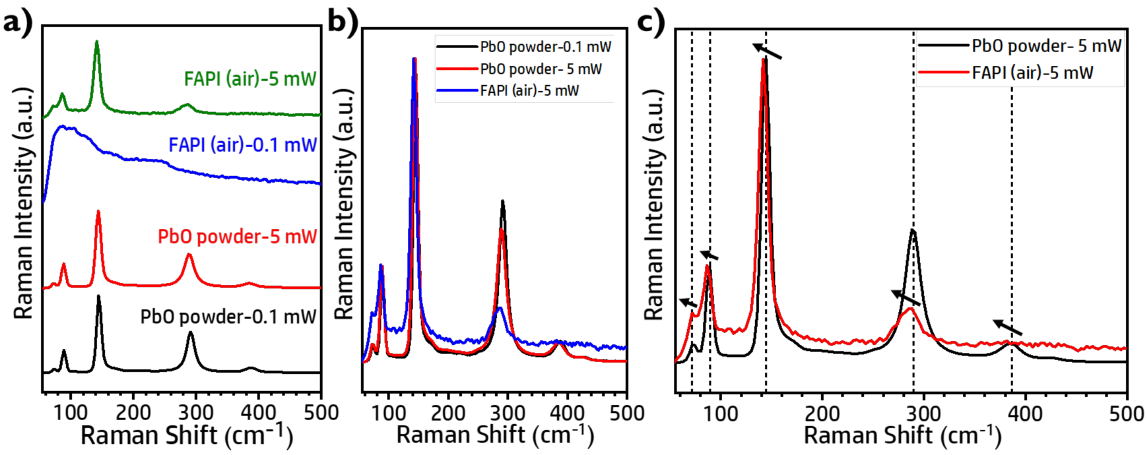

Figure S28. The Raman spectra of FAPI (air) and PbO crystal powder; a, b) at various power intensity values (0.1 and 5 mW); c) the comparative plot at 5 mW power intensity and the arrows highlighted the perovskite mode softening seen in each of the vibrations compared at 74, 89, 144, 291, and 387 cm^{-1}, respectively.

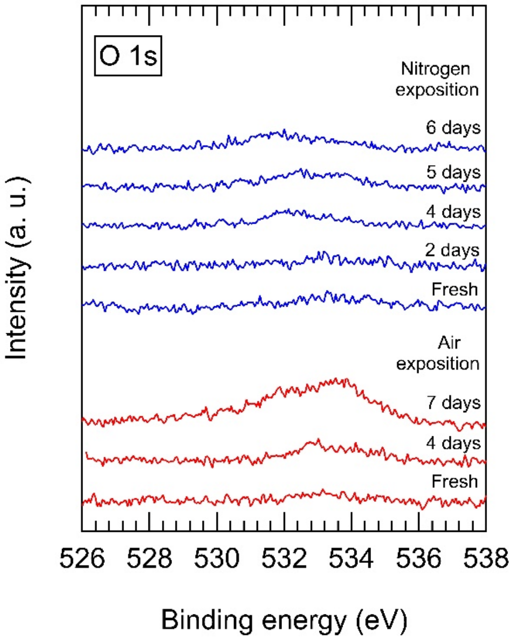

Figure S29. High energy resolution X-ray photoelectron spectra of the O 1s core level measured in FAPI (air) -red curves- and FAPI (N_2) -blue curves- films with the different aging degrees, which ranges between fresh and 7 days aged films.

Table S7. The photovoltaic performance characteristics of reference MACl (air) and MACl+1 mg/mL (air), fabricated at RH=40-60%. The average photovoltaic characteristics of solar cells including the standard deviations of the open-circuit voltage (*V*oc), short-circuit current density (*J*sc), and fill factor (FF). The champion cell efficiency of MACl (air), and MACl+1 mg/mL (air) are given in the brackets. The average statistical distribution is based on 20 solar cells on different substrates.

Sample	V_{oc} (V)	J_{sc} (mA cm^{-2})	FF	PCE (%)
**With MACl (air)**	1.02±0.01 **(1.03)**	22.87±0.49 **(23.55)**	0.76±0.01 **(0.78)**	17.98±0.53 **(18.83)**
**With MACl + 1 mg/mL (air)**	1.03±0.02 **(1.06)**	22.94±0.42 **(23.56)**	0.77±0.01 **(0.78)**	18.25±0.57 **(19.38)**

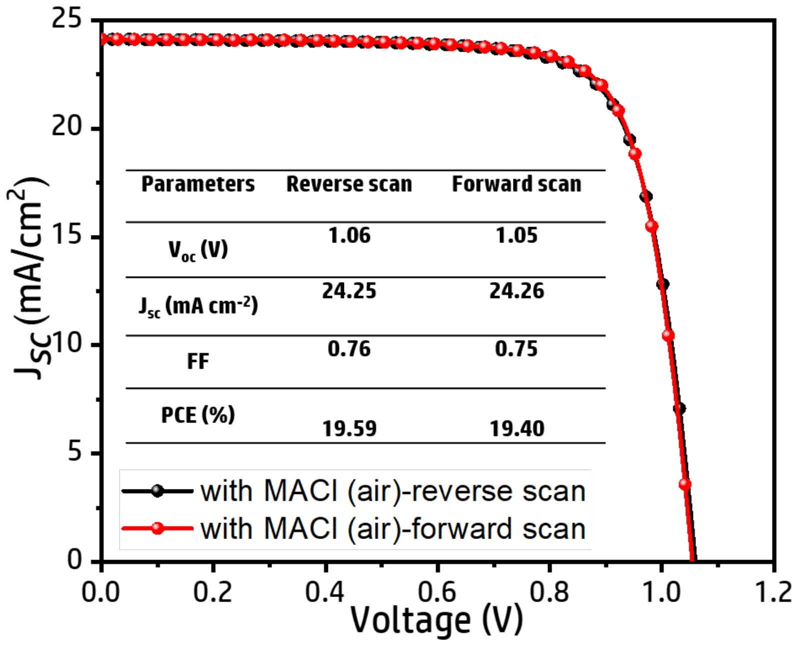

Figure S30. a) The current-voltage (J-V) curves of reference MACl (air) device recorded at forward and reverse bias scan direction (-0.02 V to 1.2 V and vice versa) with a scan rate of 10 mV/s. The devices were fabricated at a lower relative humidity of ~30%.

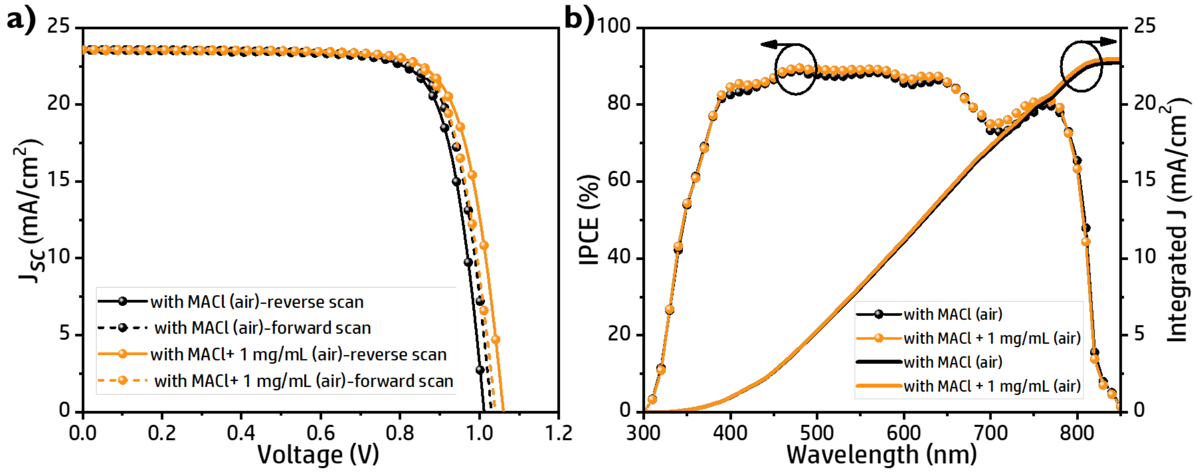

Figure S31. a) The current-voltage (J-V) curves of reference MACl (air) and MACl+1 mg/mL (air) device recorded at forward and reverse bias scan direction (-0.02 V to 1.2 V and vice versa) with a scan rate of 10 mV/s. b) IPCE spectra and integrated current density of reference MACl (air) and MACl+1 mg/mL (air) devices.

Table S8. The photovoltaic parameters of perovskite solar cells based on reference MACl (air) and MACl+1 mg/mL (air) under 1 sun illumination (AM 1.5 G, 1000 Wm^{-2}). All the devices are recorded at forward and reverse bias scan direction (-0.02 V to 1.2 V and vice versa) with a scan rate of 10 mV/s. The champion cell efficiency is given in the brackets and the average statistical distribution is based on 20 solar cells.

Sample	Scan direction	V_{oc} (V)	J_{sc} (mA cm^{-2})	FF	PCE (%)	Best PCE (%)
**With MACl (air)**	reverse scan	1.02±0.01 **(1.03)**	22.87±0.49 **(23.55)**	0.76±0.01 **(0.78)**	17.98±0.53 **(18.83)**	18.83
	forward scan	1.01±0.01 **(1.01)**	22.60±0.30 **(23.53)**	0.75±0.02 **(0.77)**	17.56±0.40 **(18.52)**	18.52
**With MACl + 1 mg/mL (air)**	reverse scan	1.03±0.02 **(1.06)**	22.94±0.42 **(23.56)**	0.77±0.01 **(0.78)**	18.25±0.57 **(19.38)**	19.38
	forward scan	1.02±0.02 **(1.04)**	22.80±0.40 **(23.57)**	0.76±0.01 **(0.77)**	18.16±0.20 **(19.15)**	19.15

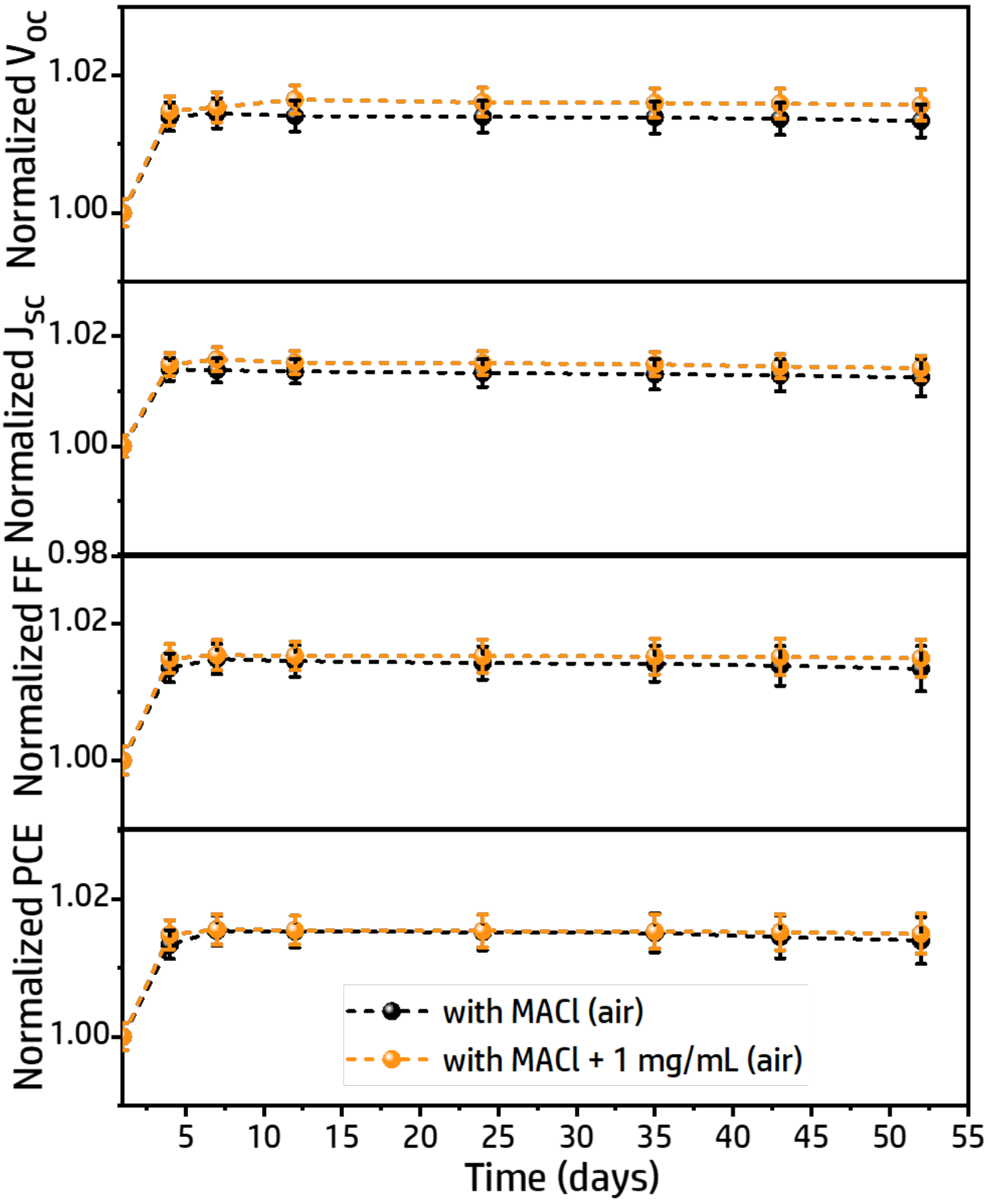

Figure 32. The stability comparison of the unencapsulated reference MACl (air) and MACl+1 mg/mL (air) devices (the data obtained from the average of 5 different devices), the normalized photovoltaic parameters like; a) open-circuit voltage (V_{OC}); b) short-circuit current density (J_{SC}); c) fill factor (FF), and d) power conversion efficiency (PCE), samples were stored with a relative humidity of 40% and 25°C, at dark conditions.

References

1. Ngo, T. T.; Masi, S.; Mendez, P. F.; Kazes, M.; Oron, D.; Seró, I. M., PbS quantum dots as additives in methylammonium halide perovskite solar cells: the effect of quantum dot capping. *Nanoscale Advances* **2019,** *1* (10), 4109-4118.
2. Lee, J.-W.; Dai, Z.; Lee, C.; Lee, H. M.; Han, T.-H.; De Marco, N.; Lin, O.; Choi, C. S.; Dunn, B.; Koh, J.; Di Carlo, D.; Ko, J. H.; Maynard, H. D.; Yang, Y., Tuning Molecular Interactions for Highly Reproducible and Efficient Formamidinium Perovskite Solar Cells via Adduct Approach. *Journal of the American Chemical Society* **2018,** *140* (20), 6317-6324.

3. Wang, G.; Wang, L.; Qiu, J.; Yan, Z.; Tai, K.; Yu, W.; Jiang, X., Fabrication of efficient formamidinium perovskite solar cells under ambient air via intermediate-modulated crystallization. *Solar Energy* **2019,** *187*, 147-155.
4. Yoo, S.-M.; Yoon, S. J.; Anta, J. A.; Lee, H. J.; Boix, P. P.; Mora-Seró, I., An Equivalent Circuit for Perovskite Solar Cell Bridging Sensitized to Thin Film Architectures. *Joule* **2019,** *3* (10), 2535-2549.
5. Masi, S.; Echeverría-Arrondo, C.; Salim, K. M. M.; Ngo, T. T.; Mendez, P. F.; López-Fraguas, E.; Macias-Pinilla, D. F.; Planelles, J.; Climente, J. I.; Mora-Seró, I., Chemi-Structural Stabilization of Formamidinium Lead Iodide Perovskite by Using Embedded Quantum Dots. *ACS Energy Letters* **2020,** *5* (2), 418-427.
6. Sánchez-Godoy, H. E.; Erazo, E. A.; Gualdrón-Reyes, A. F.; Khan, A. H.; Agouram, S.; Barea, E. M.; Rodriguez, R. A.; Zarazúa, I.; Ortiz, P.; Cortés, M. T.; Muñoz-Sanjosé, V.; Moreels, I.; Masi, S.; Mora-Seró, I., Preferred Growth Direction by PbS Nanoplatelets Preserves Perovskite Infrared Light Harvesting for Stable, Reproducible, and Efficient Solar Cells. *Advanced Energy Materials* **2020,** *10* (46), 2002422.
7. Ngo, T. T.; Suarez, I.; Sanchez, R. S.; Martinez-Pastor, J. P.; Mora-Sero, I., Single step deposition of an interacting layer of a perovskite matrix with embedded quantum dots. *Nanoscale* **2016,** *8* (30), 14379-14383.
8. Yang, Z.; Janmohamed, A.; Lan, X.; García de Arquer, F. P.; Voznyy, O.; Yassitepe, E.; Kim, G.-H.; Ning, Z.; Gong, X.; Comin, R.; Sargent, E. H., Colloidal Quantum Dot Photovoltaics Enhanced by Perovskite Shelling. *Nano Letters* **2015,** *15* (11), 7539-7543.
9. Zhang, X.; Zhang, J.; Phuyal, D.; Du, J.; Tian, L.; Öberg, V. A.; Johansson, M. B.; Cappel, U. B.; Karis, O.; Liu, J.; Rensmo, H.; Boschloo, G.; Johansson, E. M. J., Inorganic CsPbI3 Perovskite Coating on PbS Quantum Dot for Highly Efficient and Stable Infrared Light Converting Solar Cells. *Advanced Energy Materials* **2018,** *8* (6), 1702049.
10. Liu, M.; Chen, Y.; Tan, C.-S.; Quintero-Bermudez, R.; Proppe, A. H.; Munir, R.; Tan, H.; Voznyy, O.; Scheffel, B.; Walters, G.; Kam, A. P. T.; Sun, B.; Choi, M.-J.; Hoogland, S.; Amassian, A.; Kelley, S. O.; García de Arquer, F. P.; Sargent, E. H., Lattice anchoring stabilizes solution-processed semiconductors. *Nature* **2019,** *570* (7759), 96-101.
11. Kim, G.; Min, H.; Lee, K. S.; Lee, D. Y.; Yoon, S. M.; Seok, S. I., Impact of strain relaxation on performance of α-formamidinium lead iodide perovskite solar cells. *Science* **2020,** *370* (6512), 108-112.
12. Chen, J.; Park, N.-G., Causes and Solutions of Recombination in Perovskite Solar Cells. *Advanced Materials* **2019,** *31* (47), 1803019.
13. Heo, J. H.; Han, H. J.; Kim, D.; Ahn, T. K.; Im, S. H., Hysteresis-less inverted CH3NH3PbI3 planar perovskite hybrid solar cells with 18.1% power conversion efficiency. *Energy & Environmental Science* **2015,** *8* (5), 1602-1608.
